# The molecular signature of therapeutic mesenchymal stem cells exposes the architecture of the hematopoietic stem cell niche synapse

**DOI:** 10.1186/1471-2164-8-65

**Published:** 2007-03-06

**Authors:** Enrico Pedemonte, Federica Benvenuto, Simona Casazza, Gianluigi Mancardi, Jorge R Oksenberg, Antonio Uccelli, Sergio E Baranzini

**Affiliations:** 1 Department of Neurology, School of Medicine, University of California, San Francisco, CA, USA; 2 Neuroimmunology Unit, Department of Neurosciences, Ophthalmology and Genetics, University of Genoa, Italy; 3 Centre of Excellence for Biomedical Research, University of Genoa, Italy

## Abstract

**Background:**

The hematopoietic stem cells (HSCs) niche of the bone marrow is comprised of HSCs, osteoblasts, endothelial cells and a stromal component of non-hematopoietic multipotent cells of mesenchymal origin named "mesenchymal stem cells" (MSCs).

**Results:**

Here we studied the global transcriptional profile of murine MSCs with immuno-therapeutic potential and compared it with that of 486 publicly available microarray datasets from 12 other mouse tissues or cell types. Principal component analysis and hierarchical clustering identified a unique pattern of gene expression capable of distinctively classifying MSCs from other tissues and cells. We then performed an analysis aimed to identify absolute and relative abundance of transcripts in all cell types. We found that the set of transcripts uniquely expressed by MSCs is enriched in transcription factors and components of the Wnt signaling pathway. The analysis of differentially expressed genes also identified a set of genes specifically involved in the HSC niche and is complemented by functional studies that confirm the findings. Interestingly, some of these genes play a role in the maintenance of HSCs in a quiescent state supporting their survival and preventing them from proliferating and differentiating. We also show that MSCs modulate T cell functions in vitro and, upon in vivo administration, ameliorate experimental autoimmune encephalomyelitis (EAE).

**Conclusion:**

Altogether, these findings provide novel and important insights on the mechanisms of T cell function regulation by MSCs and help to cement the rationale for their application in the treatment of autoimmune diseases.

## Background

Mesenchymal stem cells (MSCs) were first isolated from bone marrow stroma and described initially as precursors for fibroblasts [1]. More recently, their multi-lineage differentiation capacity has been extensively characterized [2]. It has been previously shown that MSCs are able to differentiate into mature cells of multiple mesenchymal tissues including chondrocytes, myocytes, adipocytes and osteocytes in a process largely regulated by members of the Wnt family of proteins [3]. MSCs are also an essential component of the stromal scaffold of the bone marrow that provides physical support during hematopoiesis. Based on these findings, MSCs have been utilized to provide help for the engraftment of hematopoietic stem cells (HSCs) *in vivo *[4]. Stromal cells from the bone marrow, together with osteoblasts and endothelial cells, have been involved in the formation of the so called HSC niche [5]. This can be defined as a specialized microenvironment that precisely maintains a long-term storage of quiescent, slowly dividing HSCs by preventing their proliferation, differentiation or apoptosis [6,7].

Moreover, it has been recently shown that MSCs exert a profound modulatory effect on immune cells by means of both soluble and cell contact-dependent mechanisms [8-10]. In particular, MSCs inhibit T and B cell proliferation by halting cell cycle progression [11-13] and, under some circumstances, also rescue T cells from apoptosis (Benvenuto, F. et al., unpublished observation). In a recent experiment that highlights their therapeutic potential, *in vivo *injection of MSCs ameliorated the course of chronic progressive experimental autoimmune encephalomyelitis (EAE), the animal model of multiple sclerosis [13]. Although MSCs are able to affect a wide array of immunological functions and, as a consequence, to control the course of EAE, the underlying mechanism by which this occurs remains largely unknown. However, it is likely that both cell to cell contact and soluble factors are involved supporting T cell inhibition in a non cognate fashion. Among the latter, transforming growth factor-β1 (TGF-β1) and hepatocyte growth factor (HGF) [8], indoleamine 2,3-dioxygenase (IDO) [14], and prostaglandin E2 (PGE_2_) [15] have been proposed as consistent candidates but other studies have failed to reproduce these results [10,16]. In order to identify a new set of genes suggestive of their biological role, we used microarray technology to examine the global transcriptional profile of MSCs extracted from the bone marrow of C57BL/6J mice. We applied bioinformatics tools to analyze our results in the context of more than 400 publicly available expression datasets and identified unique features of MSCs that are likely responsible for their observed phenotype and potential therapeutic properties. Based on our experimental results we discuss the possibility that the immunoregulatory properties of MSCs may be orchestrated by the same mechanisms that are involved in the maintenance of the HSC niche.

## Results

In this study we evaluated the global transcriptional profile of immunomodulatory MSCs and conducted a bioinformatics-based analysis to identify the possible mechanisms for their unique properties. Since the process of obtaining these cells involves a laborious, multi-step cell culturing effort, prior to the transcriptional analysis we tested our MSC enrichment protocol with functional assays that tested their immunoregulatory and therapeutic functions.

### MSCs exhibit a distinctive capacity of controlling T cell proliferation and survival

**Figure 1:**
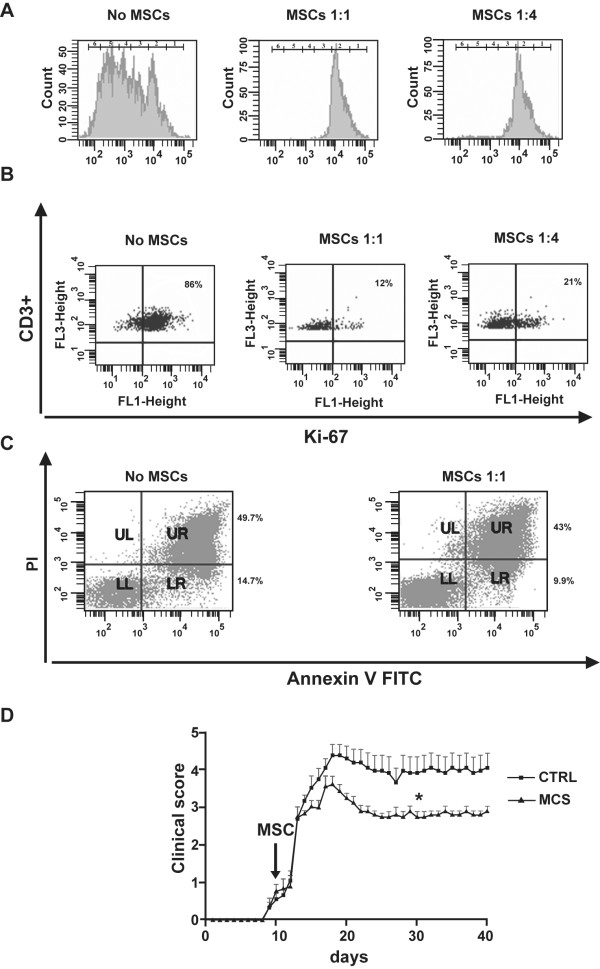
**MSCs modulate T cell functions**. (**a**) T cell stimulation leads to several cycles of cell division (left panel) while the co-culture with MSCs (middle and right panel) almost completely abrogates T cell proliferation and induces cell division arrest. (**b**) Ki67, a nuclear antigen associated with cell proliferation is downregulated on stimulated T cells when co-cultured with MSCs. (**c**) MSCs inhibit AICD as depicted by a decrease of total annexin V positive T cells (UR+LR quadrants). (**d**) Intravenous injection of MSCs ameliorates EAE. MSCs injected at day 10 post immunization, after disease onset ameliorates EAE (*p < 0.05). Arrow indicates day of MSC injection. EAE (filled triangles), controls (filled squares).

We harvested MSCs from the bone marrow of C57BL/6J mice and expanded them in culture until they reached a stable CD45-CD44+Sca1+ phenotype by FACS-analysis (data not shown). In order to test their capacity for modulating T cell functions, we added MSCs to mononuclear cells (MNCs) that had been previously activated by incubation with anti-CD3 antibodies. From as early as passage 7 in culture, MSCs inhibited T cell proliferation by inducing cell cycle arrest. This was demonstrated by the observed inhibition of CFSE incorporation (Fig. 1a) and the accumulation of T cells in the G0 phase of the cycle (Fig. 1b). When T cells are exposed to IL-2 prior to TCR engagement with an anti-CD3 mAb, they undergo apoptosis, a phenomenon known as activation induced cell death (AICD). As shown in Figure 1c, MSCs were able to rescue T cells from apoptosis as they reduced the percentage of apoptotic T cells following AICD. Finally, we examined the *in vivo *immunosuppressive potential of MSCs by injecting 1 × 10^6 ^cells into mice with MOG_35–55_-induced experimental autoimmune encephalomyelitis (EAE). The i.v. administration of 1 × 10^6 ^MSCs following disease onset resulted in a statistically significant amelioration of EAE clinical symptoms compared to PBS-injected control mice (Fig. 1d). These results strongly suggest that MSCs have a profound therapeutic effect on T cells not only by preventing their proliferation but also by supporting their survival. These findings are consistent with the dual function observed for bone marrow stromal cells, known to inhibit HSC proliferation and, at the same time, support their survival within the "niche" [4].

### Therapeutic MSCs show a unique gene expression signature

**Figure 2:**
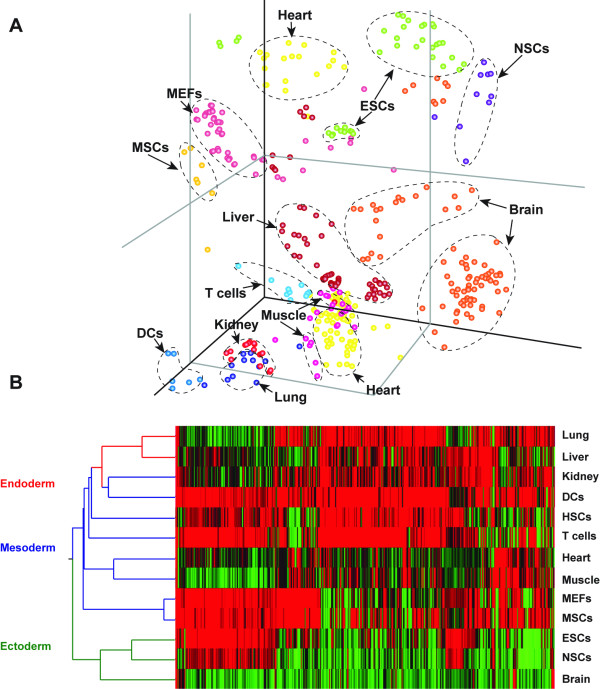
**Therapeutic MSCs show a unique gene expression signature**. (**a**) Principal component analysis of samples based on the expression of 500 genes. (**b**) Hierarchical clustering of genes and averaged samples utilizing smooth correlation metric with indication of the germinal layer of origin. MSCs: mesenchymal stem cells; MEFs: mouse embryonic fibroblasts; ESCs: embryonic stem cells; NSCs: neural stem cells; HSCs: hematopoietic stem cells; DCs: dendritic cells.

We extracted total RNA from 7 cultured MSC samples deriving from different mice and passages (see Methods) with proven immunomodulatory ability, and analyzed their gene expression profiles by hybridization onto Affymetrix Mouse Genome 430 2.0 arrays. We then compared these results to those reported in 486 publicly available platform-compatible microarrays exploring other 6 murine tissues (brain, heart, skeletal muscle, liver, kidney, lung) and 6 cell types (dendritic cells, DCs, embryonic stem cells, ESCs, embryonic fibroblasts, MEFs, neural stem cells, NSCs, HSCs, T cells). We first used principal component analysis (PCA), a dimensionality reduction technique, with the goal of testing whether the expression variance within tissues was smaller than that across tissues. In this exploratory analysis we were able to observe a recognizable segregation of samples according to the source of their RNA. The distribution of samples in PCA space was particularly pronounced for MSCs, which were clearly separated from most other tissues and cell types (Fig. 2a). Furthermore, the spread observed within principal components of MSCs was markedly lower than that observed for arrays derived from brain or ESCs. We hypothesize that although part of the observed pattern is due to sample heterogeneity (arrays from brain were obtained from several different laboratories and under many different conditions), a true biological effect is primarily responsible for the clear separation among groups of arrays. A two-way hierarchical clustering analysis confirmed the PCA results and allowed us to identify groups of genes characteristic of each tissue. In this analysis, MSC signature was readily observed when we compared the expression profile of ontogenically related cell types (Fig. 2b). Since MSCs are direct precursors of fibroblasts, it would be expected that they exhibit some degree of similarity. Interestingly, both analyses located samples from these two cell types next to each other, suggesting an overall similarity in their expression patterns. Remarkably, the unsupervised hierarchical clustering of samples resulted in a nearly perfect sorting of samples by germinal layer of origin (Fig. 2b). In addition to this, the striking similarity between ESCs and NSCs at the transcriptional level is consistent with previous reports [17]. These findings provide further evidence that the genes selected for validation are discriminatory, and that the in-silico analysis is sensitive enough to detect the characteristic signature of each cell type. Furthermore, the discriminatory power of these profiles seems to rely on a relatively small subset of genes.

### Transcripts differentially expressed by MSCs belong to the HSC niche

**Figure 3:**
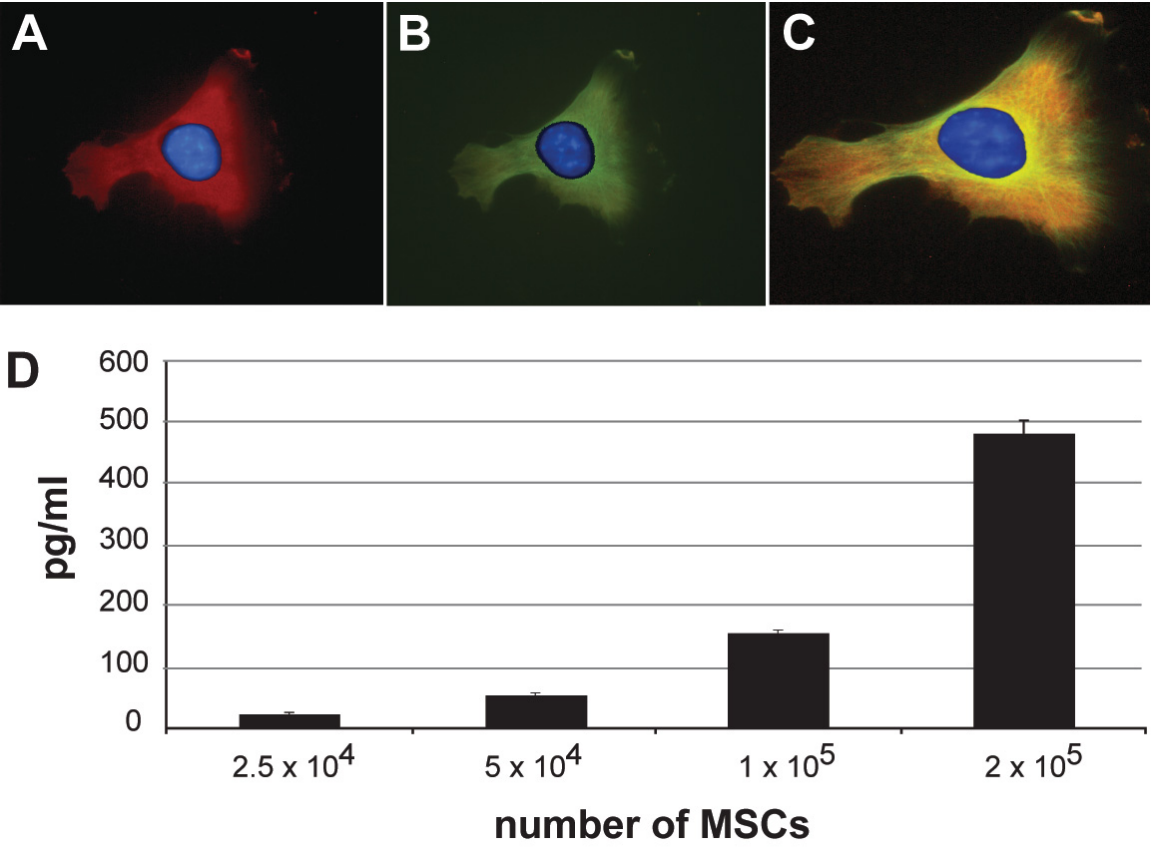
**Expression of galectin-1**. Immunocytochemistry staining of galectin-1 (red, **a**) beta-tubulin (green, **b**), and merged image (**c**). (**d**) Dosage of galectin-1 by ELISA at different concentrations of MSCs.

**Figure 4:**
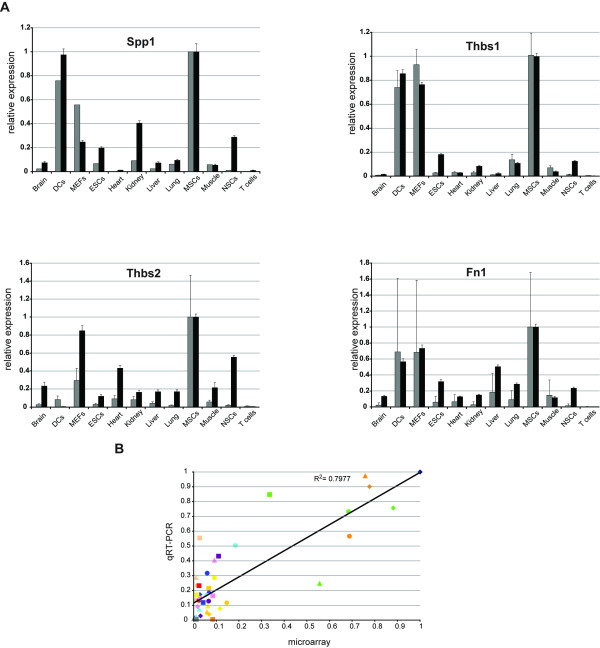
**Validation of microarray results**. **(a) **Microarray levels (grey bars) represent the signal intensity of probe-sets corresponding to the same gene (averaged when more than one present). qRT-PCR data (black bars) are obtained utilizing a GAPDH standard curve. Relative expression levels with respect to those of MSCs are shown. (**b**) Correlation between microarray (X axis) and qRT-PCR (Y axis) expression values (fold-differences versus MSCs). triangles = *Spp1*, circles = *Fn1*, diamonds = *Thbs1*, squares = *Thbs2*, violet = Heart, red = Brain, yellow = Lung, rose = Kidney, orange = DCs, green = MEFs, tan = NSCs, grey = T cells, blue = ESCs, gold = Muscle, turqoise = Liver.

In order to identify the characteristic signature of MSCs we performed two types of analyses. First, we defined differentially expressed transcripts as those expressed in all tissues but showing statistically significant differences in pair-wise comparison against MSCs. Our second analysis focused on cell "specific" genes, that is those that were detected in one but not in any other cell type. We found 403 differentially expressed genes between MSCs and any given tissue or cell type in our panel [See Additional file 1]. Among these, galectin-1 (*Lgals1*) was the highest expressed transcript in MSCs, a finding that correlates well with previous reports [18,19]. We next sought to verify whether high expression of *Lgals1 *was also a characteristic of MSCs at the protein level. Indeed, the intracellular presence of *Lgals1 *colocalizing with MSC cytoskeleton was detected by immunostaining and visualized by confocal microscopy (Fig. 3). We then confirmed that *Lgals1 *is also secreted by MSCs as demonstrated by its presence in MSC supernatants measured by ELISA (Fig. 3). These results provide further support for a biological role of the previously observed transcriptional signature of MSCs. Among the transcripts with the highest signal intensities, we were able to detect several hematopoiesis related molecules. These transcripts code mostly for secreted proteins that play a role within the HSC niche such as fibronectin-1 (*Fn1*), osteopontin (*Spp1*), chemokine C-X-C motif ligand-12 (*Cxcl12*), thrombospondin-1 (*Thbs1*), thrombospondin-2 (*Thbs2*), transforming growth factor-β2 (*Tgfb2*), angiopoietin-1 (*Angpt1*), insulin-like growth factor binding protein-4 (*Igfbp4*), fibroblast growth factor-7 (*Fgf7*), secreted frizzled-related protein-1 (*Sfrp1*), secreted frizzled-related protein-2 (*Sfrp2*), dickkopf-3 (*Dkk3*), vascular cell adhesion molecule-1 (*Vcam1*), and bone morphogenetic protein receptor type 1a (*Bmpr1a*). The expression of 4 representative genes from this list was validated by quantitative RT-PCR using commercially obtained RNA representing most tissues previously analyzed by microarray (Fig. 4). In general, there was good agreement between the expression of the tested genes in MSCs and other tissues and cell types (R^2 ^= 0.7977), thus confirming our observations. Unexpectedly, the expression of these genes was shared not only by MSCs and MEFs, but also by DCs.

### Transcripts uniquely expressed by MSCs are mostly transcription factors and genes downstream of the Wnt signaling pathway

In order to identify tissue-specific transcripts we next computed a list with all the probes called "present" in the tissue of interest and "absent" in all other tissues (see Methods) [See Additional file 2]. Notably, the percentage of genes identified as "specific" increased proportionally with the ontological complexity of each tissue or cell type (with the exception of heart). This is consistent with the idea that a terminally differentiated cell needs to activate the expression of a higher number of genes in order to accomplish its specific tasks. As expected from a highly specialized parenchymatous tissue, brain was the sample with the highest percentage of specific genes. We next compiled a list with the 381 resulting specific MSC genes and ordered them according to signal intensity [See Additional file 3].

Interestingly, a sizable proportion of MSC specific genes were either transcription factors or molecules belonging to or downstream of the Wnt signaling pathway [3,20] (*Sfrp1*, *Tcf3*, *Rarg*, *Cspg2*, *Dvl2*, *Axin1*). Wnt proteins are highly conserved, lipid-modified secreted ligands that influence multiple processes in animal development. In particular, they mediate important decisions between proliferative self-renewal and differentiation of stem cells. The average signal intensity of these genes was also markedly lower than the intensity of most differentially expressed genes suggesting that these transcripts are expressed at low levels.

### Gene Ontology (GO) statistical analysis of genes differentially and uniquely expressed by MSCs

**Figure 5:**
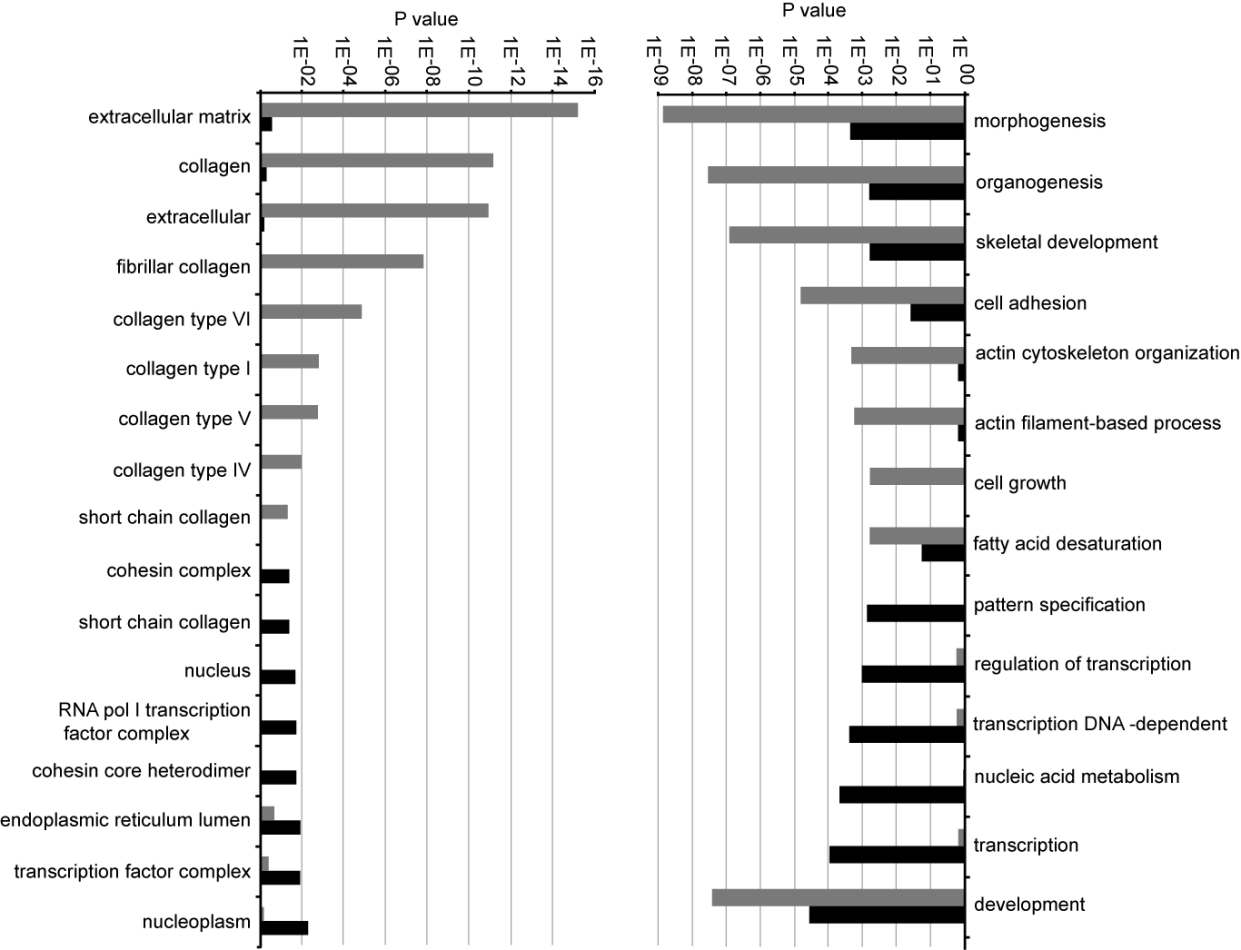
**GO statistical analysis**. GO statistical analysis of the most enriched Cell Component (left) and Biological Process (right) categories for differentially expressed genes (grey bars) and specific genes (black bars).

In order to integrate expression profiles into a functional framework we performed a GO-based statistical analysis. When we analyzed the 403 differentially expressed genes, we found that *extracellular matrix *and *extracellular *were among the first three statistically significant GO Cell Component categories, while *cell adhesion *was among the first five most enriched Biological Process classes (Fig. 5). This suggests that the most abundantly expressed transcripts were mainly secreted proteins, a significant part of which are involved in cell-cell contact. Conversely, when we analyzed the list of "specific" genes, they appeared to be primarily located in the *nucleoplasm *and mainly involved in *development *and *transcription *processes (Fig. 5). This strongly suggests that the list of "specific" genes is composed mainly by low level-expressed transcription factors and molecules involved in development (i.e. Wnt pathway).

## Discussion

Overall, this report provides a transcriptional map of immunomodulatory MSCs through global RNA profiling and bioinformatics-based comparative analysis with several other mouse tissues and cell types. By analyzing arrays from the same platform (Affymetrix 430) we were able to perform statistical analyses combining results across different laboratories. Thus, we overcame the limitation of comparisons across different platforms, generally regarded as problematic from a biological perspective because the measurements may represent different physical quantities [21]. Conversely, the approach used in this study offers a more precise assessment of the true degree of differential expression and helps refine the list of candidate genes warranting biological validation.

By applying principal component and hierarchical clustering analyses, we identified a distinctive gene expression profile for MSCs. MSC gene expression profile appeared in fact to be characteristic and substantially homogeneous. One of the most significant findings of this analysis was the nearly perfect sorting of tissues by germinal layer of origin utilizing an unsupervised classification. As expected, being MEFs derived from MSCs, their expression profile was highly similar. Furthermore, previous studies have shown that cultured MEFs maintain some of the multipotency of MSCs [22]. Surprisingly, MSCs and DCs displayed a similar profile as well, a finding further confirmed by qRT-PCR analysis. We speculate this could be due to the fact that both MSCs and DCs are involved in the determination of bystander cell fate, respectively HSCs and T cells, by cell-cell contact-dependent mechanisms.

When focusing on the list of differentially expressed genes we found that many of them had been reported in previous transcriptional studies of MSCs, thus validating the sensitivity and reproducibility of our approach [18,23]. Among MSC specific genes, several proteins belonging to the Wnt signaling pathway or Wnt-regulated genes (*Sfrp1*, *Dvl2*, *Axin1*, *Tcf3*, *Rarg*, *Cspg2*, *Efnb2*) were detected. Wnt proteins have been previously reported to promote myogenesis [24], inhibit chondrogenesis [25] and adipogenesis [26] and to have a dual effect on osteogenesis [27]. Some of the most abundantly expressed genes are also inhibitors (*Sfrp1*, *Sfrp2*, *Dkk3*) or act downstream (*Thbs1*, *Thbs2*, Cyr61, *Sema3a*, *Sfrp2*) of the Wnt signaling pathway [20]. Among the differentially expressed genes we were also able to find most recently described molecules involved in stem cell renewal such as *Dkk3 *[28] and *Serpinf1 *(PEDF) [29]. Altogether, these results are consistent with the involvement of Wnt signaling in mesenchymal lineage specification.

We also used GO-based functional classification to characterize both differentially expressed and MSC specific genes. This analysis confirmed our primary observation that differentially expressed transcripts were significantly enriched in molecules present in the extracellular space and involved in cell adhesion process. On the other hand, MSC specific genes were mainly nucleoplasm-located transcription factors involved in development. Importantly, the observation that these transcription factors are expressed at low levels is consistent with most recent findings about the biology of ESCs. These cells have been recently shown to express low levels of transcription factors by means of the polycomb group proteins and by specific chromatin modification patterns thus keeping them poised for activation during development [30]. It can be speculated that a similar mechanism may be working in further committed stem cells such as MSCs.

The combined transcriptional and comparative analyses presented here allowed us to identify several secreted molecules that may be critical in determining cell functions. Several of these molecules have been previously reported to play a pivotal role in the constitution of the HSC niche synapse [5] or as being expressed by hematopoiesis-supportive stromal cells [31] [See additional file 4]. For example, angiopoietin-1 (*Angpt1*) is considered one of the major regulators of blood vessel stability [32]and, in contrast with angiopoietin-2 (*Angpt2*), it seems to exert a potent anti-inflammatory effect [33]. Moreover, *Angpt1 *and its receptor endothelial-specific receptor tyrosine kinase(*Tek*) are known key components of the HSC niche, namely of the cell-cell signaling between osteoblasts and HSCs. Furthermore, *Angpt1 *is likely to be one of the most important molecules in the induction of the "quiescent" state of the HSCs, by enhancing their tight adhesion to osteoblasts, inhibiting cell division, arresting cells in the G0 phase of the cell cycle and promoting cell survival under myelosuppressive stress [34].

Osteopontin (*Spp1*) is a highly acidic phosphoprotein with pleiotropic effects, including regulation of inflammation, cell adhesion and angiogenesis [35]. In addition, it has recently been demonstrated to be a pivotal molecule in limiting the size of the HSC pool [36]. Similarly, it has also been shown that the thrombin-cleaved form of *Spp1 *promotes the quiescence of HSCs by exerting a profound suppression of proliferation of HSCs without inducing apoptosis [37]. Interestingly, *Spp1*-deficient mice developed a milder form of EAE, and activated T cells from these animals produced less IFNγ and more IL-10 than their wild type counterparts consistently with the role of a pro-inflammatory molecule [38]. Although an elevated *Spp1 *expression in therapeutic MSCs is in apparent contradiction with its previously reported proinflammatory role, its pleiotropism and ubiquitousness suggest that it may interact with different receptors at different times and through different regulatory networks, thus preventing a straightforward characterization of its biological functions.

Thrombospondin (*Thbs*)-1 and -2 are extracellular matrix proteins that share several effects on cell growth, adhesion, survival and differentiation. In particular, *Thbs1*, secreted by DCs, has been demonstrated to inhibit both TCR signaling and T cell proliferation, mainly acting through the CD47 receptor [39]. In addition, *Thbs1 *has also been described as part of the HSC niche [40]. Both *Thbs1 *and, to a lesser extent, *Thbs2 *have been studied as inhibitors of endothelial growth [41]. On the other hand, *Thbs2 *has been described as an autocrine inhibitor of proliferation secreted by MSCs which acts through cell-cycle inhibition without the induction of apoptosis [42].

Fibronectin-1 (*Fn1*) is another major component of the bone marrow stroma. The interaction of *Fn1 *with β1-integrins, VLA-5 in particular, has been shown to be a negative regulator of HSC proliferation by preventing them from entering the cell cycle [43].

Galectin-1 (*Lgals1*) is an endogenous lectin characterized by its affinity for beta-galactosides which has been demonstrated to exert an immunosuppressive effect on T cells through both apoptotic and non-apoptotic mechanisms [44]. This molecule has been shown to be expressed by hematopoietic-related tissues [23] and to inhibit HSC growth particularly at high concentrations [45]. Most recently, *Lgals1 *has been shown to play a role within the NSC niche [46]. We were able to confirm MSC expression of this molecule both by ELISA and immunocytochemistry assays.

Finally, semaphorins are molecules that were originally identified as mediators of axon guidance but have also been described as part of the immunological synapse [47]. Semaphorin 3A (*Sema3a*), in particular, has also been recently demonstrated to mediate T cell proliferation inhibition by arresting T cells in the G0/G1 phase of the cell cycle [48].

## Conclusion

Here we have shown that MSCs arrest T cells in the G0 phase of the cell cycle. These results are in line with previous reports showing that MSCs induce T cell [12], B cell [11] and dendritic cell division arrest anergy [49]. Such effect not only does not depend on the induction of apoptosis on immune cells [13] but is accompanied by an anti-apoptotic effect on target cells, when they are overstimulated. Although this dual in-vitro effect of MSCs on T cells appears counterintuitive as the mechanism responsible for ameliorating EAE, we speculate that MSCs may exert their immunoregulatory action by virtue of their anti-proliferative effect, while their anti-apoptotic action is mostly beneficial for the survival and protection of neural cells. Recent data seem to support this hypothesis [50].

**Figure 6:**
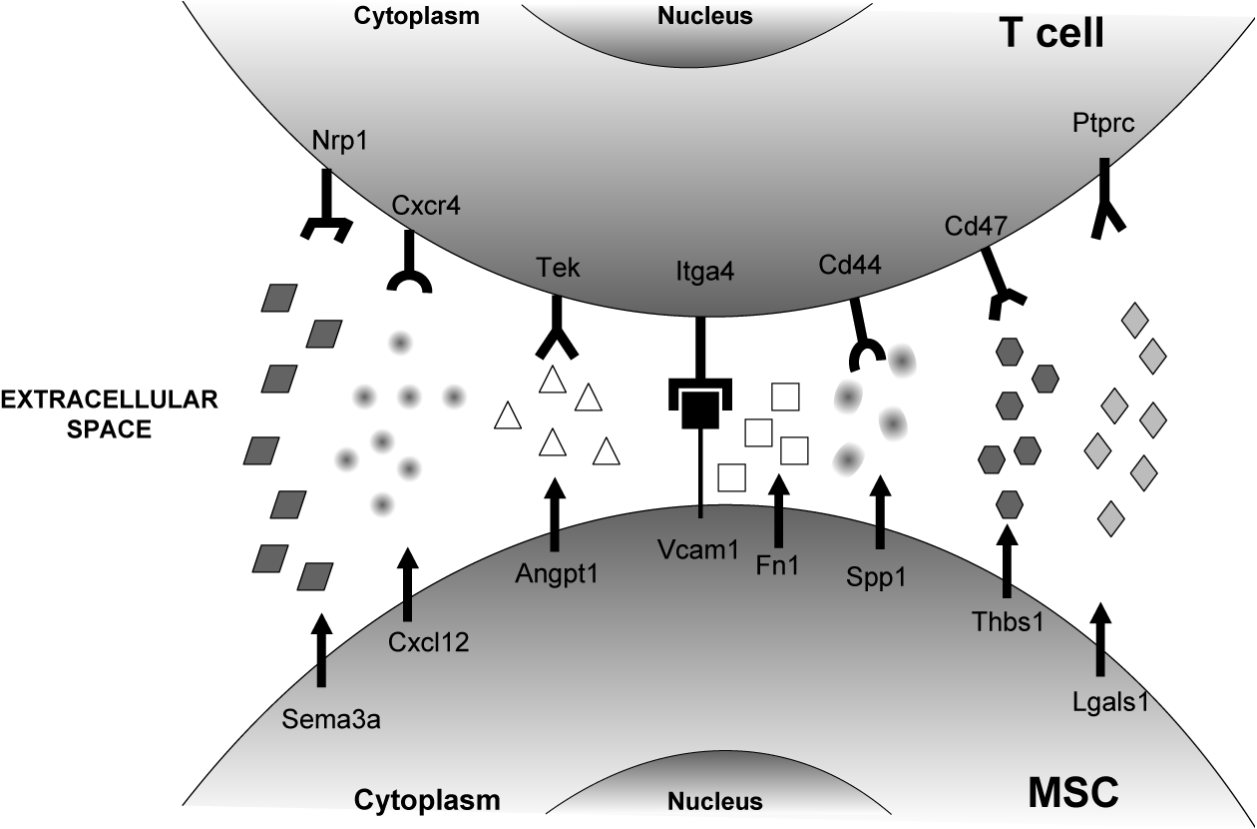
**Model of MSC-T cell interactions**. *Fn1*: fibronectin-1, *Spp1*: osteopontin, *Cxcl12*: chemokine C-X-C motif ligand-12, *Thbs1*: thrombospondin-1, *Angpt1*: angiopoietin-1, *Vcam1*: vascular cell adhesion molecule-1, *Lgals1*: galectin-1, Sema3a: semaphorin-3A, Nrp1: neuropilin-1, Cxcr4: chemokine C-X-C motif receptor-4, Tek: endothelial-specific receptor tyrosine kinase, Itga4: integrin alpha-4 (VLA-4), Ptprc: protein tyrosine phosphatase receptor type-C (CD45).

Our data clearly shows that immunomodulatory MSCs abundantly express several molecules also secreted by osteoblasts in the context of the HSC niche synapse where they may keep HSCs quiescent while supporting their survival. In addition, some of these molecules belong to the protein class of matricellular proteins (*Spp1*, *Thbs1*, *Thbs2*, *Sparc*), namely extracellular matrix proteins that, rather than serving a primary structural role, mediate both tissue morphogenesis and homeostasis modulating cell-matrix and cell-cell interactions and other important processes such as cell growth, angiogenesis and inflammation. This observation, together with the similarities we found between MSC and DC transcriptomes, highlights the relevance of macromolecular assemblies at the interface between MSCs and surrounding cells. We hypothesize that, the interactions between MSCs and HSCs resemble those described for the immunological synapse thus leading to quiescence both of proliferating HSCs and immune cells. Therefore, given the analogies between the effect exerted by osteoblasts on HSCs and by MSCs on immune cells, we propose that the same bystander mechanisms may play a role in both types of cell-cell interaction (Fig. 6). At the same time, interaction between MSCs, their extracellular matrix components and differentiating somatic cells is an established mechanism within stem cell niches throughout the body [51]. Based on our experimental results, we also hypothesize that MSCs represent fundamental niche cells within the bone marrow and potentially other stem cell niches as well. In particular, when administered *in vivo*, they could participate in the formation and modulation of the recently named "atypical ectopic perivascular niches" in the context of inflammatory disorders of the CNS [52]. Overall, our data shed further light into the biology of MSCs by proposing several candidate molecules as potential mediators both of the HSC niche-supportive and immunomodulatory activities of MSCs.

## Methods

### MSC isolation and expansion

MSCs were isolated and expanded from C57BL/6J mice as described elsewhere [13]. Briefly, bone marrow from 6- to 8-week-old mice was flushed out of tibias and femurs and plated in murine MesenCult (StemCell Technologies, Vancouver, BC) at the concentration of 0.3 to 0.4 × 10^6^cells/cm^2^. When 80% confluent, adherent cells were trypsinized, harvested and then replated in order to expand them until a homogeneous population was obtained in about 4 to 5 weeks. The phenotype of MSCs was analyzed by cell surface analysis using the FACS Canto flow cytometer as previously described [13]. The following monoclonal antibodies directed against mouse surface markers anti-CD45 cytochrome C (CyC), anti-stem cell antigen-1 (Sca-1) phycoerythrin (PE), unconjugated anti-CD9, anti-CD3 CyC, anti-CD4 peridinin chlorophyll-alpha protein (PerCP), anti-CD73 PE were purchased from Pharmingen/Becton Dickinson. The phenotype of MSCs was routinely verified by flow cytometry using the following combination of antibodies: CD11b FITC/CD34 PE/CD45 CyC and CD9 FITC/Sca-1 PE/CD45 CyC.

### In vitro and in vivo immunomodulatory experiments

Mononuclear cells (MNCs) were obtained from the spleen and the lymph nodes of healthy C57BL/6J mice and isolated by ficoll gradient (Lympholite, Cedarlane, Hornby, ON). MNCs were then utilized for standard proliferation assays as described elsewhere [13]. T cell proliferation was evaluated by CFSE incorporation by dividing CD3 positive cells. In order to study the phase of T cell cycle, we evaluated the intracellular expression of Ki67, a nuclear antigen associated with cell proliferation, present throughout the active cell cycle (G1, S, G2 and M phases) but absent in resting cells (G0). For the induction of apoptosis, 2 × 10^6 ^MNCs were pretreated with 100 U/ml of IL-2 for three days and were subsequently overstimulated with 5 μg/ml of anti-CD3 for 4 days in the presence or absence of MSCs at 1:1 ratio in a 96 well plate. The number of apoptotic T cells that underwent activation induced cell death (AICD), has been studied analyzing the number of annexin V positive cells by flow cytometry gating on CD3 positive cells.

EAE experiments were carried out as described elsewhere [13]. On day fifteen post-immunization, usually considered the peak of disease, mice received intravenous 1 × 10^6 ^MSCs diluted in PBS. Controls received intravenous injections of an equal volume of PBS on the same day. Clinical score was assigned daily according to the standard and validated scale from 0 to 5. Disease incidence, onset, and maximum score were recorded for each mouse and expressed as mean ± SD.

### Gene expression profiling

Seven immunomodulatory MSC cultures obtained between the 7^th ^and the 12^th ^passage in culture were analyzed for gene expression. Total RNA was isolated from confluent cultured cells using TRIzol reagent and further cleaned up with RNeasy Mini kit (Qiagen). RNA was then amplified and labeled for hybridization onto Affymetrix Mouse Genome 430 2.0 arrays according to the manufacturer's instructions (Affymetrix, Inc. Santa Clara, CA). All seven samples were hybridized washed and scanned at the TGen microarray core facility (Phoenix, AZ).

### Microarray data analysis

In addition to the MSC arrays generated in our lab, we also analyzed other 486 Affymetrix Mouse Genome 430A, 430B and 430 2.0 arrays corresponding to 6 murine tissues (brain, n = 90; heart n = 73; kidney n = 21; liver n = 72; and skeletal muscle n = 58). We also analyzed public arrays from 6 murine cell types (T cells n = 14; dendritic cells, DCs, n = 6; embryonic stem cells, ESCs, n = 60; embryonic fibroblasts, MEFs n = 44; neural stem cells, NSCs n = 20, and hematopoietic stem cells, HSCs n = 4). All the publicly available expression sets (including our own) can be downloaded from the Gene Expression Omnibus website [53], with their respective GEO accession numbers [See Additional file 5]. The raw intensity of individual samples was imported into BRB-Array Tools (Biometric Research Branch, National Institutes of Health, Bethesda, MD). Expression data was normalized by computing the median of the intensity distribution frequency for each array. Only genes showing at least a 4-fold change from that gene's median value on 20% or more of the arrays were considered for subsequent analysis. For statistical significance of all class comparisons, we performed F tests with a nominal value of *p *< 0.0001, and corrected the for multiple comparisons using the FDR (false discovery rate) method. For principal component analysis and hierarchical clustering, the raw intensity data were exported to GeneSpring GX software (Agilent Technologies Inc, Palo Alto, CA). To define the list of "specific" genes we utilized 399 arrays which expressed the probe-set call, namely an absolute analysis that indicates if the transcript is present (P), absent (A) or marginal (M). We then defined a transcript as detected when it was called "P" or "M" in at least 70% of the arrays from every tissue or cell type. We imported these data into Microsoft Access to obtain a list of "specific" genes, namely genes detected only in MSC arrays and not in other tissues or cell types.

### Gene ontology analysis

Selected gene lists were annotated using open source EASE version 2.0 gene ontology (GO) analysis software [54]. For each GO group, EASE computed the number of genes represented on the microarray for that group and the statistical significance *p *value for each gene in the group. These *p *values reflect differential expression among classes and were computed based on Fisher's exact probability test controlled by FDR. We considered the abundance of a GO category significantly different if either significance level was < 0.01.

### Quantitative RT-PCR

Murine total RNA from liver, brain, heart, lung and kidney was purchased from Ambion Inc (Austin, TX). Murine skeletal muscle total RNA was purchased from Stratagene (La Jolla, CA). Primary MEFs were purchased from StemCell Technologies (Vancouver, BC). RNA was isolated from murine T cells, DCs, ESCs, NSCs and MEFs using RNeasy Mini kit (Qiagen, Valencia, CA) and cleaned up with RNase-Free DNase Set (Qiagen, Valencia, CA). Total RNA was reverse transcribed using Multiscribe reverse transcriptase (Applied Biosystems, Foster City, CA). The TaqMan gene expression arrays and the TaqMan Universal Master Mix were used according to the manufacturer's recommendations (Applied Biosystems). Measurement and analysis of gene expression were performed using the ABI Prism 7900HT Sequence Detection System software (Applied Biosystems). Content of cDNA samples was obtained by utilizing the GAPDH relative standard curve method as described elsewhere [55].

### Direct Enzyme-Linked Immunosorbent Assay for galectin-1 (*Lgals1*)

Supernatants from MSC cultures were repeatedly spinned down at 3500 g through 10 and 30 kDa cut-off filters in Centricon tubes (YM-10, YM-30 Millipore Corporation, Bedford, MA) to increase detection of *Lgals1 *(around 14 kDa) and incubated in MaxiSorp plates (Nunc, Rochester, NY) at 4°C overnight. Upon washing, *Lgals1 *was directly detected by incubation with an anti-*Lgals1 *monoclonal biotinylated antibody (R&D Systems, Minneapolis, MN) for 2 hours at room temperature and, subsequently, with streptavidin-HRP (R&D Systems, Minneapolis, MN) for 20 minutes at room temperature. Following the addition of the TMB substrate, optical densities were measured at 450 nm in an ELISA reader (Metertech INC, Taiwan).

### Immunofluorescence

MSCs were plated in chamber slides (Lab Tek II, chamber slide system, Nalge Nunc, Naperville, IL) at the concentration of 10^4 ^cells/well and then incubated at 37°C in 5% CO_2 _for three days. Next, cells were fixed in 4% PFA, washed in PBS with 1% BSA (Sigma Aldrich, St. Louis, MO) at room temperature for 5 minutes and blocked with a blocking buffer (20% donkey serum and 0.05% tween 20 in PBS with 1% BSA) for 30 minutes at room temperature. In each well we added the mouse anti-*Lgals1 *antibody (2 μg/ml, R&D Systems Minneapolis, MN) and mouse anti-beta-tubulin antibody (2 μg/ml, Chemicon, Temecula CA) and the slides incubated in a humid chamber overnight at 4°C. These antibodies were revealed respectively with an Alexa 546 conjugated secondary antibody (1 μg/ml, Molecular Probes, Eugene, OR) and with Alexa 488 conjugated secondary antibody (1 μg/ml, Molecular Probes, Eugene, OR) for 30 minutes at room temperature, followed by washing in PBS. The chamber slides were mounted with "VECTASHIELD Mounting Medium with DAPI" (Vector Laboratories, Burlingame CA). The chamber slides were analyzed by confocal microscopy (Leica TCS SP2 AOBS Systems, Leica microsystems, Heidelberg GmbH).

## Authors' contributions

EP: Carried out molecular and bioinformatics experiments, and wrote the paper.

FB: Participated in immunological experiments.

SC: Carried out animal work and immunostainings.

GM: Participated in the design of the study. Provided research materials.

JRO: Conceived the study and participated in drafting the paper.

AU: Participated in study design and statistical analysis. Provided research materials.

SEB: Conceived the study and participated in its design and coordination. Participated in bioinformatics and statistical analysis, and wrote the paper.

All authors read and approved the final manuscript.

## Supplementary Material

Additional file 1 Genes differentially expressed in MSC. Genes associated with the HSC niche are highlighted. Click here for file

Additional file 2 "Specific" probesets. Samples are represented in circles with different sizes proportional to the absolute amount of probesets detected only in the correspondent tissue or cell type. On Y axis, the proportion of "specific" probesets among all the detected probesets per tissue/cell type is shown. Click here for file

Additional file 3 MSC "specific" genes. Genes related to or downstream of the Wnt signaling pathway are highlighted Click here for file

Additional file 4 Candidate molecules for MSC biological activities. A compilation of molecules potentially regulating the activity of MSCs. Click here for file

Additional file 5 Expression datasets used in this article. Description of microarray experiments used in this article. For each experiment, the laboratory of origin, the tissue and the Affymetrix platform are indicated. Click here for file
