# Supplementary material for: The molecular signature of therapeutic mesenchymal stem cells exposes the architecture of the hematopoietic stem cell niche synapse
Source: BMC Genomics. 2007 Mar 6;8:65. doi: 10.1186/1471-2164-8-65 (PMC1821333; doi:10.1186/1471-2164-8-65)
Supplement: Additional file 1 — Genes differentially expressed in MSC. Genes associated with the HSC niche are highlighted. [file 1471-2164-8-65-S1.pdf]

Additional File 1. Genes differentially expressed in MSC.

Probe set	Gene symbol	Description	Mean intensity	p value
**1455439_a_at**	**Lgals1**	**lectin, galactose binding, soluble 1**	**69051.3**	**< 1e-07**
1450857_a_at	Col1a2	procollagen, type I, alpha 2	47324.4	< 1e-07
1450641_at	Vim	vimentin	46066.5	< 1e-07
**1419573_a_at**	**Lgals1**	**lectin, galactose binding, soluble 1**	**45522.7**	**< 1e-07**
1437889_x_at	Bgn	biglycan	44032.5	< 1e-07
1448323_a_at	Bgn	biglycan	41917.2	< 1e-07
**1426642_at**	**Fn1**	**fibronectin 1**	**38397.3**	**< 1e-07**
1416405_at	Bgn	biglycan	37362.5	< 1e-07
1437165_a_at	Pcolce	procollagen C-endopeptidase enhancer protein	37323.1	< 1e-07
1416589_at	Sparc	secreted acidic cysteine rich glycoprotein	35360.4	< 1e-07
1421375_a_at	S100a6	S100 calcium binding protein A6 (calcyclin)	34619.8	< 1e-07
1416121_at	Lox	lysyl oxidase	34460.7	< 1e-07
1438118_x_at	Vim	vimentin	34108.2	< 1e-07
1422437_at	Col5a2	procollagen, type V, alpha 2	33958.9	< 1e-07
1419091_a_at	Anxa2	annexin A2	33710	< 1e-07
1438403_s_at			32235.8	< 1e-07
1423669_at	Col1a1	procollagen, type I, alpha 1	32226.4	< 1e-07
**1449254_at**	**Spp1**	**secreted phosphoprotein 1**	**31605.2**	**< 1e-07**
1448392_at	Sparc	secreted acidic cysteine rich glycoprotein	31489	< 1e-07
1423754_at	Ifitm3	interferon induced transmembrane protein 3	31467.5	< 1e-07
1423110_at	Col1a2	procollagen, type I, alpha 2	31270.3	< 1e-07
1439374_x_at		Transcribed locus, strongly similar to NP_080239 ribosomal proteinS10	30464.3	< 1e-07
**1448823_at**	**Cxcl12**	**chemokine (C-X-C motif) ligand 12**	**28813.9**	**< 1e-07**
1420820_at	2900073G15Rik	RIKEN cDNA 2900073G15 gene	28597.8	< 1e-07
1456292_a_at	Vim	vimentin	26852.4	< 1e-07
1448416_at	Mgp	matrix Gla protein	26670.7	< 1e-07
1448228_at	Lox	lysyl oxidase	26508.1	< 1e-07
1427883_a_at	Col3a1	procollagen, type III, alpha 1	25497.3	< 1e-07
1456312_x_at	Gsn	gelsolin	25323.8	< 1e-07
1460287_at	Timp2	tissue inhibitor of metalloproteinase 2	24668	< 1e-07
1416122_at	Ccnd2	cyclin D2	23973.1	< 1e-07
1448433_a_at	Pcolce	procollagen C-endopeptidase enhancer protein	23716.1	< 1e-07
1416256_a_at	Tubb5	tubulin, beta 5	23055.9	< 1e-07
1415822_at	Scd2	stearoyl-Coenzyme A desaturase 2	22984.2	< 1e-07
1456733_x_at	Serpinh1	serine (or cysteine) peptidase inhibitor, clade H, member 1	22546.9	< 1e-07
1416740_at	Col5a1	procollagen, type V, alpha 1	22191.2	< 1e-07
1450843_a_at	Serpinh1	serine (or cysteine) peptidase inhibitor, clade H, member 1	21965.9	< 1e-07
1448590_at	Col6a1	procollagen, type VI, alpha 1	21745	< 1e-07
1452217_at	Ahnak	AHNAK nucleoprotein (desmoyokin)	21716.2	< 1e-07
1416168_at	Serpinf1	serine (or cysteine) peptidase inhibitor, clade F, member 1	20615.6	< 1e-07
1437171_x_at	Gsn	gelsolin	20485.2	< 1e-07
1417308_at	Pkm2	pyruvate kinase, muscle	20441	< 1e-07
1451112_s_at	Dap	death-associated protein	20015.4	< 1e-07
1450981_at	Cnn2	calponin 2	19949.5	< 1e-07

1415812_at	Gsn	gelsolin	19721.7	< 1e-07
**1421811_at**	**Thbs1**	**thrombospondin 1**	**19627.2**	**< 1e-07**
1454677_at	Timp2	tissue inhibitor of metalloproteinase 2	19565.9	< 1e-07
1416039_x_at	Cyr61	cysteine rich protein 61	19284.6	< 1e-07
1417460_at	Ifitm2	interferon induced transmembrane protein 2	19249.9	< 1e-07
1433883_at	Tpm4	tropomyosin 4	19035.5	< 1e-07
1416221_at	Fstl1	follistatin-like 1	18590.7	< 1e-07
1452250_a_at	Col6a2	procollagen, type VI, alpha 2	18545.7	< 1e-07
1448179_at	Usmg5	upregulated during skeletal muscle growth 5	17548.7	< 1e-07
1448259_at	Fstl1	follistatin-like 1	16559.1	< 1e-07
**1460302_at**	**Thbs1**	**thrombospondin 1**	**16449.8**	**< 1e-07**
1436991_x_at	Gsn	gelsolin	15929.9	< 1e-07
1424770_at	Cald1	caldesmon 1	15811.9	< 1e-07
1450138_a_at	Serpinb6a	serine (or cysteine) peptidase inhibitor, clade B, member 6a	15608.2	< 1e-07
1435800_a_at	Csda	cold shock domain protein A	15589.7	< 1e-07
1434745_at	Ccnd2	cyclin D2	15479.6	< 1e-07
1430127_a_at	Ccnd2	cyclin D2	15435.5	< 1e-07
1433662_s_at	Timp2	tissue inhibitor of metalloproteinase 2	14866.7	< 1e-07
1420827_a_at	Ccng1	cyclin G1	14813.6	< 1e-07
1416136_at	Mmp2	matrix metallopeptidase 2	14792.5	< 1e-07
1423407_a_at	Fbln2	fibulin 2	14504.1	< 1e-07
1455956_x_at	Ccnd2	cyclin D2	14456.9	< 1e-07
1437992_x_at	Gja1	gap junction membrane channel protein alpha 1	14435.6	< 1e-07
1439364_a_at	Mmp2	matrix metallopeptidase 2	14317.5	< 1e-07
1426677_at	Flna	filamin, alpha	13623.8	< 1e-07
1423606_at	Postn	periostin, osteoblast specific factor	13620.5	< 1e-07
**1417574_at**	**Cxcl12**	**chemokine (C-X-C motif) ligand 12**	**13537.6**	**< 1e-07**
1456380_x_at	Cnn3	calponin 3, acidic	13444.7	< 1e-07
1423584_at	Igfbp7	insulin-like growth factor binding protein 7	13265.9	< 1e-07
1418188_a_at	Malat1	metastasis associated lung adenocarcinoma transcript 1 (non-coding RNA)	13218.4	< 1e-07
1433575_at	Sox4	SRY-box containing gene 4 (Sox4), mRNA	12375.7	< 1e-07
1417065_at	Egr1	early growth response 1	12332.1	< 1e-07
1447643_x_at	Snai2	snail homolog 2 (Drosophila)	12186.1	< 1e-07
1450757_at	Cdh11	cadherin 11	11942.9	< 1e-07
1424131_at	Col6a3	procollagen, type VI, alpha 3	11937.2	< 1e-07
1434976_x_at	Eif4ebp1	eukaryotic translation initiation factor 4E binding protein 1	11888.7	< 1e-07
1424768_at	Cald1	caldesmon 1	11808.7	< 1e-07
1453724_a_at	Serpinf1	serine (or cysteine) peptidase inhibitor, clade F, member 1	11779.2	< 1e-07
1423418_at	Fdps	farnesyl diphosphate synthetase	11632.8	< 1e-07
1416855_at	Gas1	growth arrest specific 1	11248.5	< 1e-07
1449110_at	Rhob	ras homolog gene family, member B	11173	< 1e-07
1416529_at	Emp1	epithelial membrane protein 1	11115.1	< 1e-07
1448613_at	Ecm1	extracellular matrix protein 1	11024.5	< 1e-07
1422438_at	Ephx1	epoxide hydrolase 1, microsomal	10921.2	< 1e-07
1423790_at	Dap	death-associated protein	10805	< 1e-07
1452035_at	Col4a1	procollagen, type IV, alpha 1	10804.9	< 1e-07
1427918_a_at	Rhoq	ras homolog gene family, member Q	10785.9	< 1e-07
1422441_x_at	Cdk4	cyclin-dependent kinase 4	10525.7	< 1e-07
1436363_a_at	Nfix	nuclear factor I/X	10513.1	< 1e-07
1418004_a_at	1810009M01Rik	RIKEN cDNA 1810009M01 gene	10372.2	< 1e-07

**1437405_a_at**	**Igfbp4**	**insulin-like growth factor binding protein 4**	**10241.3**	**< 1e-07**
1419662_at	Ogn	osteoglycin	10038.6	< 1e-07
1451095_at	Asns	asparagine synthetase	10028	< 1e-07
1454757_s_at	D12Ertd647e	DNA segment, Chr 12, ERATO Doi 647, expressed	9977.8	< 1e-07
1417562_at	Eif4ebp1	eukaryotic translation initiation factor 4E binding protein 1	9665.3	< 1e-07
**1438405_at**	**Fgf7**	**fibroblast growth factor 7**	**9587.2**	**< 1e-07**
**1437406_x_at**	**Igfbp4**	**insulin-like growth factor binding protein 4**	**9493.1**	**< 1e-07**
1425811_a_at	Csrp1	cysteine and glycine-rich protein 1	9436.7	< 1e-07
1449002_at	Phlda3	pleckstrin homology-like domain, family A, member 3	9412.5	< 1e-07
1423824_at	5031439A09Rik	RIKEN cDNA 5031439A09 gene	9394	< 1e-07
1419835_s_at	Plec1	plectin 1	9344.6	< 1e-07
1438133_a_at	Cyr61	cysteine rich protein 61	9280.9	< 1e-07
1451012_a_at	Csda	cold shock domain protein A	9275.9	< 1e-07
1415800_at	Gja1	gap junction membrane channel protein alpha 1	9117.5	< 1e-07
1419663_at	Ogn	osteoglycin	9079.8	< 1e-07
1423078_a_at	Sc4mol	sterol-C4-methyl oxidase-like	9011.6	< 1e-07
1427262_at	Xist	inactive X specific transcripts	8994.7	< 1e-07
1417011_at	Sdc2	syndecan 2	8898.7	< 1e-07
1449335_at	Timp3	tissue inhibitor of metalloproteinase 3	8894	< 1e-07
1417133_at	Pmp22	peripheral myelin protein	8843.9	< 1e-07
1456739_x_at	Armcx2	armadillo repeat containing, X-linked 2	8613.1	< 1e-07
1450637_a_at	Aebp1	AE binding protein 1	8433.7	< 1e-07
1416498_at	Ppic	peptidylprolyl isomerase C	8412.8	< 1e-07
1428842_a_at	Ngfrap1	nerve growth factor receptor (TNFRSF16) associated protein 1	8402.3	< 1e-07
1419665_a_at	Nupr1	nuclear protein 1	8310	< 1e-07
1424874_a_at	Ptbp1	polypyrimidine tract binding protein 1	8240.5	< 1e-07
1450016_at	Ccng1	cyclin G1	7961.1	< 1e-07
1424394_at	MGI:2149786	selenoprotein M	7911	< 1e-07
1436364_x_at	Nfix	nuclear factor I/X	7844.5	< 1e-07
1416101_a_at	Hist1h1c	histone 1, H1c	7809.6	< 1e-07
1416554_at	Pdlim1	PDZ and LIM domain 1 (elfin)	7792	< 1e-07
1415823_at	Scd2	stearoyl-Coenzyme A desaturase 2	7537	< 1e-07
1424099_at	2310016C16Rik	RIKEN cDNA 2310016C16 gene	7492.4	< 1e-07
1437325_x_at	Aldh18a1	aldehyde dehydrogenase 18 family, member A1	7460.3	< 1e-07
1427256_at	Cspg2	chondroitin sulfate proteoglycan 2	7409.5	< 1e-07
1424114_s_at	Lamb1-1	laminin B1 subunit 1	7336.6	< 1e-07
1423484_at	Bicc1	bicaudal C homolog 1 (Drosophila)	7262.4	< 1e-07
1418072_at	Hist1h2bc	histone 1, H2bc	7252.7	< 1e-07
1417090_at	Rcn1	reticulocalbin 1	7228.1	< 1e-07
1451064_a_at	Psat1	phosphoserine aminotransferase 1	7223.4	< 1e-07
1415993_at	Sqle	squalene epoxidase	7201.5	< 1e-07
1417962_s_at	Ghr	growth hormone receptor	7169.6	< 1e-07
1433768_at	2410003B16Rik	RIKEN cDNA 2410003B16 gene	7149.1	< 1e-07
1426794_at	Ptprs	protein tyrosine phosphatase, receptor type, S	7113.3	< 1e-07
1433966_x_at	Asns	asparagine synthetase	7078.9	< 1e-07
1415943_at	Sdc1	syndecan 1	7049.8	< 1e-07
1434020_at	Pdap1	PDGFA associated protein 1	7029.5	< 1e-07
1451446_at	Antxr1	anthrax toxin receptor 1	6965	< 1e-07
1452787_a_at	Hrmt1l2	heterogeneous nuclear ribonucleoproteins methyltransferase-like 2 (S. cerevisiae)	6961.9	< 1e-07
1436944_x_at	4933439C20Rik	RIKEN cDNA 4933439C20 gene /// phosphatidylserine decarboxylase	6844	< 1e-07

1417012_at	Sdc2	syndecan 2	6808.8	< 1e-07
1449632_s_at	Fkbp10	FK506 binding protein 10	6800.2	< 1e-07
1416572_at	Mmp14	matrix metallopeptidase 14 (membrane-inserted)	6714.8	< 1e-07
1416687_at	Plod2	procollagen lysine, 2-oxoglutarate 5-dioxygenase 2	6662.1	< 1e-07
1437279_x_at	Sdc1	syndecan 1	6625.5	< 1e-07
1428316_a_at	Fundc2	FUN14 domain containing 2	6611.6	< 1e-07
1422603_at	Rnase4	ribonuclease, RNase A family 4	6514.2	< 1e-07
1454607_s_at	Psat1	phosphoserine aminotransferase 1	6486.3	< 1e-07
1424800_at	Enah	enabled homolog (Drosophila)	6474	< 1e-07
1436520_at	AI450948	expressed sequence AI450948	6427.4	< 1e-07
1421144_at	Rpgrip1	retinitis pigmentosa GTPase regulator interacting protein 1	6360.8	< 1e-07
1438945_x_at	Gja1	gap junction membrane channel protein alpha 1	6354.8	< 1e-07
1416749_at	Htra1	HtrA serine peptidase 1	6206.9	< 1e-07
1436970_a_at	Pdgfrb	platelet derived growth factor receptor, beta polypeptide	6200.5	< 1e-07
1419089_at	Timp3	tissue inhibitor of metalloproteinase 3	6180.3	< 1e-07
1438251_x_at	Htra1	HtrA serine peptidase 1	6174.8	< 1e-07
1450047_at	Hs6st2	heparan sulfate 6-O-sulfotransferase 2	6172.7	< 1e-07
1418773_at	Fads3	fatty acid desaturase 3	6097.1	< 1e-07
1416129_at	Errfi1	ERBB receptor feedback inhibitor 1	6020.8	< 1e-07
1448229_s_at	Ccnd2	cyclin D2	5998.4	< 1e-07
1418571_at	Tnfrsf12a	tumor necrosis factor receptor superfamily, member 12a	5995.8	< 1e-07
1425528_at	Prrx1	paired related homeobox 1	5831.4	< 1e-07
1438312_s_at	Ltbp3	latent transforming growth factor beta binding protein 3	5753.3	< 1e-07
1423493_a_at	Nfix	nuclear factor I/X	5714.8	< 1e-07
1434875_a_at	Hmgn3	high mobility group nucleosomal binding domain 3	5665.5	< 1e-07
1423915_at	Olfml2b	olfactomedin-like 2B	5607	< 1e-07
1419666_x_at	Nupr1	nuclear protein 1	5603.7	< 1e-07
1423725_at	Pls3	plastin 3 (T-isoform)	5587.9	< 1e-07
1426348_at	Col4a1	procollagen, type IV, alpha 1	5485	< 1e-07
1448894_at	Akr1b8	aldo-keto reductase family 1, member B8	5459.8	< 1e-07
1445597_s_at	Hrasls3	HRAS like suppressor 3	5449.7	< 1e-07
1420831_at	Qscn6	quiescin Q6	5368.4	< 1e-07
1448605_at	Rhoc	ras homolog gene family, member C	5294.7	< 1e-07
1425810_a_at	Csrp1	cysteine and glycine-rich protein 1	5268.9	< 1e-07
1441137_at	Bicc1	Bicaudal C homolog 1 (Drosophila) (Bicc1), mRNA	5261.5	< 1e-07
1418674_at	Osmr	oncostatin M receptor	5249.2	< 1e-07
1418572_x_at	Tnfrsf12a	tumor necrosis factor receptor superfamily, member 12a	5242.5	< 1e-07
1450040_at	Timp2	tissue inhibitor of metalloproteinase 2	5228.9	< 1e-07
1437247_at	Fosl2	fos-like antigen 2	5114.1	< 1e-07
1418538_at	Kdelr3	KDEL (Lys-Asp-Glu-Leu) endoplasmic reticulum protein retention receptor 3	5105.3	< 1e-07
1425927_a_at	Atf5	activating transcription factor 5	5000.1	< 1e-07
1451969_s_at	Parp3	poly (ADP-ribose) polymerase family, member 3	4894.3	< 1e-07
1436729_at	2600003E23Rik	RIKEN cDNA 2600003E23 gene	4874.2	< 1e-07
1415951_at	Fkbp10	FK506 binding protein 10	4806.4	< 1e-07
1449183_at	Comt	catechol-O-methyltransferase	4625.1	< 1e-07
1419254_at	Mthfd2	methylenetetrahydrofolate dehydrogenase (NAD+ dependent), methenyltetrahydrofolate cyclohydrolase	4574.4	< 1e-07
1424882_a_at	2510015F01Rik	RIKEN cDNA 2510015F01 gene	4573.5	< 1e-07
1416608_a_at	BC004004	cDNA sequence BC004004	4491.1	< 1e-07
1455893_at	Rspo2	R-spondin 2 homolog (Xenopus laevis)	4466.6	< 1e-07

1433795_at	Tgfbr3	transforming growth factor, beta receptor III	4424.4	< 1e-07
1456084_x_at	Fmod	fibromodulin	4406.7	< 1e-07
1421694_a_at	Cspg2	chondroitin sulfate proteoglycan 2	4331	< 1e-07
1416414_at	Emilin1	elastin microfibril interfacer 1	4330.8	< 1e-07
1422738_at	Ddr2	discoidin domain receptor family, member 2	4293.4	< 1e-07
1448494_at	Gas1	growth arrest specific 1	4278.2	< 1e-07
1436659_at	Dcamkl1	Double cortin and calcium/calmodulin-dependent protein kinase-like 1	4275.7	< 1e-07
1419456_at	Dcxr	dicarbonyl L-xylulose reductase	4241.4	< 1e-07
1451532_s_at	Steap1	six transmembrane epithelial antigen of the prostate 1	4219.2	< 1e-07
1455627_at	Col8a1	procollagen, type VIII, alpha 1	4197.8	< 1e-07
1432517_a_at	Nnmt	nicotinamide N-methyltransferase	4187.9	< 1e-07
1417009_at	C1r	complement component 1, r subcomponent	4117.2	< 1e-07
1416612_at	Cyp1b1	cytochrome P450, family 1, subfamily b, polypeptide 1	4063.4	< 1e-07
1424041_s_at	C1s	complement component 1, s subcomponent	4032.9	< 1e-07
1417045_at	Bid	BH3 interacting domain death agonist	3983.2	< 1e-07
1415780_a_at	Armcx2	armadillo repeat containing, X-linked 2	3979.1	< 1e-07
1420924_at	Timp2	tissue inhibitor of metalloproteinase 2	3978.4	< 1e-07
1434423_at	0	0	3939.2	< 1e-07
1421045_at	Mrc2	mannose receptor, C type 2	3898.3	< 1e-07
1432331_a_at	Prrx2	paired related homeobox 2	3870.1	< 1e-07
1453851_a_at	Gadd45g	growth arrest and DNA-damage-inducible 45 gamma	3840	< 1e-07
1426210_x_at	Parp3	poly (ADP-ribose) polymerase family, member 3	3825.7	< 1e-07
1416411_at	Gstm2	glutathione S-transferase, mu 2	3805.5	< 1e-07
1438650_x_at	Gja1	gap junction membrane channel protein alpha 1	3773.5	< 1e-07
1450700_at	Cdc42ep3	CDC42 effector protein (Rho GTPase binding) 3	3764.9	< 1e-07
1450871_a_at	Bcat1	branched chain aminotransferase 1, cytosolic	3761.4	< 1e-07
1428636_at	Steap2	six transmembrane epithelial antigen of prostate 2	3700.6	< 1e-07
1424801_at	Enah	enabled homolog (Drosophila)	3648.4	< 1e-07
1424086_at	D9Ucla1	DNA segment, Chr 9, University of California at Los Angeles 1	3642.4	< 1e-07
1424938_at	Steap1	six transmembrane epithelial antigen of the prostate 1	3605.5	< 1e-07
1425985_s_at	Masp1	mannan-binding lectin serine peptidase 1	3467.5	< 1e-07
1428418_s_at	3110050N22Rik	RIKEN cDNA 3110050N22 gene	3466.9	< 1e-07
1418532_at	Fzd2	frizzled homolog 2 (Drosophila)	3466.2	< 1e-07
1423835_at	Zfp503	zinc finger protein 503	3374.4	< 1e-07
1419978_s_at	D10Ertd610e	DNA segment, Chr 10, ERATO Doi 610, expressed	3373.9	< 1e-07
1418533_s_at	Fzd2	frizzled homolog 2 (Drosophila)	3367.2	< 1e-07
1451501_a_at	Ghr	growth hormone receptor	3278	< 1e-07
1418326_at	Slc7a5	solute carrier family 7 (cationic amino acid transporter, y+ system), member 5	3265.2	< 1e-07
1448593_at	Wisp1	WNT1 inducible signaling pathway protein 1	3214.5	< 1e-07
1432198_at			3202.6	< 1e-07
1435527_at	1500041O16Rik	RIKEN cDNA 1500041O16 gene	3199.2	< 1e-07
1454877_at	Sertad4	SERTA domain containing 4	3164.3	< 1e-07
1421344_a_at	Jub	ajuba	3124.8	< 1e-07
1434314_s_at	Rab11fip5	RAB11 family interacting protein 5 (class I)	3119.9	< 1e-07
1448594_at	Wisp1	WNT1 inducible signaling pathway protein 1	3104.6	< 1e-07
1436994_a_at	Hist1h1c	histone 1, H1c	3001.2	< 1e-07
1426910_at	Pawr	PRKC, apoptosis, WT1, regulator	2971.4	< 1e-07
1423516_a_at	Nid2	nidogen 2	2958.5	< 1e-07
1425476_at	Col4a5	procollagen, type IV, alpha 5	2926.7	< 1e-07
1431475_a_at	Hoxa10	homeo box A10	2874.8	< 1e-07

1415801_at	Gja1	gap junction membrane channel protein alpha 1	2869.8	< 1e-07
1426865_a_at	Ncam1	neural cell adhesion molecule 1	2811.9	< 1e-07
1449334_at	Timp3	tissue inhibitor of metalloproteinase 3	2810	< 1e-07
1418673_at	Snai2	snail homolog 2 (Drosophila)	2793	< 1e-07
1451413_at	Cast	calpastatin	2731	< 1e-07
1416632_at	Mod1	malic enzyme, supernatant	2689.2	< 1e-07
1415944_at	Sdc1	syndecan 1	2680.8	< 1e-07
1431777_a_at	Hmgn3	high mobility group nucleosomal binding domain 3	2656	< 1e-07
1449325_at	Fads2	fatty acid desaturase 2	2645.7	< 1e-07
1417408_at	F3	coagulation factor III	2623.5	< 1e-07
1449551_at	Myo1c	myosin IC	2538.5	< 1e-07
1419031_at	Fads2	fatty acid desaturase 2	2535.7	< 1e-07
1438042_at	Shox2	short stature homeobox 2	2523.8	< 1e-07
1448501_at	Tspan6	tetraspanin 6	2436.8	< 1e-07
1442116_at	Gm1012	gene model 1012, (NCBI)	2414.6	< 1e-07
1418440_at	Col8a1	procollagen, type VIII, alpha 1	2411.5	< 1e-07
1428492_at	Glipr2	GLI pathogenesis-related 2	2349.5	< 1e-07
1425526_a_at	Prrx1	paired related homeobox 1	2347.4	< 1e-07
1430979_a_at	Prdx2	peroxiredoxin 2	2323.6	< 1e-07
1430780_a_at	Pmm1	phosphomannomutase 1	2295.6	< 1e-07
1428417_at	3110050N22Rik	RIKEN cDNA 3110050N22 gene	2279.6	< 1e-07
1420901_a_at	Hk1	hexokinase 1	2261	< 1e-07
1415877_at	Dpysl3	dihydropyrimidinase-like 3	2231.7	< 1e-07
1422561_at	Adamts5	a disintegrin-like and metallopeptidase with thrombospondin type 1 motif 5 (aggrecanase-2)	2213.8	< 1e-07
1435972_at	Cast	calpastatin	2186.7	< 1e-07
1417130_s_at	Angptl4	angiopoietin-like 4	2182.9	< 1e-07
1424113_at	Lamb1-1	laminin B1 subunit 1	2122.3	< 1e-07
1421002_at	Angptl2	angiopoietin-like 2	2112.8	< 1e-07
1427912_at	Cbr3	carbonyl reductase 3	2104.7	< 1e-07
1448326_a_at	Crabp1	cellular retinoic acid binding protein I	2102.2	< 1e-07
1449350_at	Osr1	odd-skipped related 1 (Drosophila)	2097.4	< 1e-07
1448201_at	Sfrp2	secreted frizzled-related sequence protein 2	2089.7	< 1e-07
1430522_a_at	Vamp5	vesicle-associated membrane protein 5	2028.6	< 1e-07
1418981_at	Casp12	caspase 12	2019.7	< 1e-07
1424942_a_at	Myc	myelocytomatosis oncogene	2018.8	< 1e-07
1426010_a_at	Epb4.1l3	erythrocyte protein band 4.1-like 3	2018.1	< 1e-07
1416166_a_at	Prdx4	peroxiredoxin 4	1929.4	< 1e-07
1455642_a_at	Tspan17	tetraspanin 17	1913.2	< 1e-07
1419157_at	Sox4	SRY-box containing gene 4	1891.1	< 1e-07
1455299_at	1700110N18Rik	RIKEN cDNA 1700110N18 gene	1886.8	< 1e-07
1424556_at	Pycr1	pyrroline-5-carboxylate reductase 1	1881.5	< 1e-07
1448777_at	Mcm2	minichromosome maintenance deficient 2 mitotin (S. cerevisiae)	1871.8	< 1e-07
1427884_at	Col3a1	procollagen, type III, alpha 1	1863.9	< 1e-07
1419209_at	Cxcl1	chemokine (C-X-C motif) ligand 1	1860.7	< 1e-07
1418670_s_at			1833.5	< 1e-07
**1423250_a_at**	**Tgfb2**	**transforming growth factor, beta 2**	**1825.8**	**< 1e-07**
1451985_at	Lrrk1	leucine-rich repeat kinase 1	1781.8	< 1e-07
1416123_at	Ccnd2	cyclin D2	1773.1	< 1e-07
1419156_at	Sox4	SRY-box containing gene 4	1747.5	< 1e-07
1434748_at	Ckap2	cytoskeleton associated protein 2	1727.4	< 1e-07

1451289_at	Dcamkl1	double cortin and calcium/calmodulin-dependent protein kinase-like 1	1672.9	< 1e-07
1442542_at	Eya4	eyes absent 4 homolog (Drosophila)	1634.9	< 1e-07
1423350_at	Socs5	suppressor of cytokine signaling 5	1630	< 1e-07
1418569_at	Fblim1	filamin binding LIM protein 1	1603.4	< 1e-07
1437218_at	Fn1	fibronectin 1	1585.1	< 1e-07
1448656_at	Cacnb3	calcium channel, voltage-dependent, beta 3 subunit	1577.6	< 1e-07
1452244_at	6330406I15Rik	RIKEN cDNA 6330406I15 gene	1561.6	< 1e-07
1429974_at	Tbx18	T-box18	1543.2	< 1e-07
1435120_at		Transcribed locus	1535.8	< 1e-07
1424917_a_at	D11Ertd498e	DNA segment, Chr 11, ERATO Doi 498, expressed	1530.3	< 1e-07
1434089_at	Synpo	synaptopodin	1517.6	< 1e-07
1434340_at			1517	< 1e-07
**1417312_at**	**Dkk3**	**dickkopf homolog 3 (Xenopus laevis)**	**1483.7**	**< 1e-07**
1434678_at	Mbnl3	muscleblind-like 3 (Drosophila)	1439.7	< 1e-07
1426538_a_at	Trp53	transformation related protein 53	1439.1	< 1e-07
1429987_at	9930013L23Rik	RIKEN cDNA 9930013L23 gene	1437.3	< 1e-07
1439066_at	Angpt1	angiopoietin 1	1426	< 1e-07
1455978_a_at	Matn2	matrilin 2	1423.3	< 1e-07
1416613_at	Cyp1b1	cytochrome P450, family 1, subfamily b, polypeptide 1	1420.8	< 1e-07
1439151_at	Msrb3	methionine sulfoxide reductase B3	1418.9	< 1e-07
1430111_a_at	Bcat1	branched chain aminotransferase 1, cytosolic	1409.7	< 1e-07
1418733_at	Twist1	twist gene homolog 1 (Drosophila)	1396.1	< 1e-07
1449154_at	Col11a1	procollagen, type XI, alpha 1	1389.3	< 1e-07
1423311_s_at	Tpbg	trophoblast glycoprotein	1375.7	< 1e-07
1417234_at	Mmp11	matrix metallopeptidase 11	1365.2	< 1e-07
1455160_at	2610203C20Rik	RIKEN cDNA 2610203C20 gene	1339.2	< 1e-07
1438861_at	Bnc2	basonuclin 2	1339.1	< 1e-07
1418534_at	Fzd2	frizzled homolog 2 (Drosophila)	1316.8	< 1e-07
1416376_at	1810014L12Rik	RIKEN cDNA 1810014L12 gene	1309.7	< 1e-07
1427020_at	Scara3	scavenger receptor class A, member 3	1304.5	< 1e-07
1423836_at	Zfp503	zinc finger protein 503	1296.9	< 1e-07
1450085_at	Angptl2	Angiopoietin-like 2 (Angptl2), mRNA	1288.4	< 1e-07
1459679_s_at	Myo1b	myosin IB	1250.7	< 1e-07
1424507_at	Rin1	Ras and Rab interactor 1	1203.5	< 1e-07
**1449865_at**	**Sema3a**	**sema domain, immunoglobulin domain (Ig), short basic domain, secreted, (semaphorin) 3A**	**1187.3**	**< 1e-07**
1437422_at	Sema5a	sema domain, seven thrombospondin repeats transmembrane domain and short cytoplasmic domain, (semaphorin) 5A	1186	< 1e-07
1420938_at	Hs6st2	heparan sulfate 6-O-sulfotransferase 2	1175.2	< 1e-07
1421815_at	Epdr2	ependymin related protein 2 (zebrafish)	1153	< 1e-07
1448742_at	Snai1	snail homolog 1 (Drosophila)	1131.6	< 1e-07
1450437_a_at	Ncam1	neural cell adhesion molecule 1	1124	< 1e-07
1418379_s_at	Gpr124	G protein-coupled receptor 124	1118.1	< 1e-07
1438966_x_at	Fmod	Fibromodulin (Fmod), mRNA	1113.6	< 1e-07
1451734_a_at	Dbn1	drebrin 1	1106.9	< 1e-07
1437556_at	Zfhx4	zinc finger homeodomain 4	1086.7	< 1e-07
1451583_a_at	BC025076	cDNA sequence BC025076	1073.2	< 1e-07
1431225_at	Sox11	SRY-box containing gene 11 (Sox11), mRNA	1065.7	< 1e-07
1417229_at	Capn1	calpain 1	1064.5	< 1e-07
1434286_at	Trps1	trichorhinophalangeal syndrome I (human)	1062.3	< 1e-07
1456437_x_at			1032.1	< 1e-07

1438531_at	A730054J21Rik	RIKEN cDNA A730054J21 gene	1010.4	< 1e-07
1416124_at	Ccnd2	cyclin D2	981.4	< 1e-07
1451204_at	Scara5	scavenger receptor class A, member 5 (putative)	968.2	< 1e-07
1428717_at	Scrn1	secernin 1	929.9	< 1e-07
1440305_at		0 day neonate eyeball cDNA, RIKEN full-length enriched library	913.5	< 1e-07
1456532_at	Pdgfd	platelet-derived growth factor, D polypeptide	864.7	< 1e-07
1423549_at	Slc1a4	solute carrier family 1 (glutamate/neutral amino acid transporter), member 4	836	< 1e-07
1427910_at	Cst6	cystatin E/M	803	< 1e-07
1438773_at	Steap2	PREDICTED: six transmembrane epithelial antigen of prostate 2 [Mus musculus], mRNA sequence	798.8	< 1e-07
1427263_at	Xist	inactive X specific transcripts	743.7	< 1e-07
1424270_at	Dcamkl1	double cortin and calcium/calmodulin-dependent protein kinase-like 1	735.6	< 1e-07
1421977_at	Mmp19	matrix metallopeptidase 19	730.8	< 1e-07
1417401_at	Rai14	retinoic acid induced 14	730.6	< 1e-07
1423186_at	Tiam2	T-cell lymphoma invasion and metastasis 2	714.5	< 1e-07
1424680_at	BB146404	expressed sequence BB146404	698.8	< 1e-07
1439397_at	BB164513	expressed sequence BB164513	612.6	< 1e-07
1436617_at	Cetn4	centrin 4	594.7	< 1e-07
**1450922_a_at**	**Tgfb2**	**transforming growth factor, beta 2**	**548.4**	**< 1e-07**
1457779_at	1110046J04Rik	RIKEN cDNA 1110046J04 gene	544.4	< 1e-07
1447787_x_at	Gja7	gap junction membrane channel protein alpha 7	540.5	< 1e-07
1455965_at			528.9	< 1e-07
1434776_at	Sema5a	sema domain, seven thrombospondin repeats transmembrane domain and short cytoplasmic domain (semaphorin) 5A	526.1	< 1e-07
1453769_at	2610318C08Rik	RIKEN cDNA 2610318C08 gene	515.9	< 1e-07
1449033_at	Tnfrsf11b	tumor necrosis factor receptor superfamily, member 11b (osteoprotegerin)	514.9	< 1e-07
1421624_a_at	Enah	enabled homolog (Drosophila)	492.6	< 1e-07
1435211_at	Ttc12	tetratricopeptide repeat domain 12	477.8	< 1e-07
1418872_at	Abcb1b	ATP-binding cassette, sub-family B (MDR/TAP), member 1B	474	< 1e-07
1440204_at	3110039M20Rik	RIKEN cDNA 3110039M20 gene	472.8	< 1e-07
1431430_s_at	Trim59	tripartite motif-containing 59	472.5	< 1e-07
1457827_at	9330196J05Rik	RIKEN cDNA 9330196J05 gene	461.8	< 1e-07
1460632_at	Rdh10	retinol dehydrogenase 10 (all-trans)	459.6	< 1e-07
1436877_at	Lrch2	leucine-rich repeats and calponin homology (CH) domain containing 2	456.9	< 1e-07
1454700_at	Lrfn4	leucine rich repeat and fibronectin type III domain containing 4	452.1	< 1e-07
1427640_a_at	Cbfa2t1h	CBFA2T1 identified gene homolog (human)	442.5	< 1e-07
1460206_at	Grasp	GRP1 (general receptor for phosphoinositides 1)-associated scaffold protein	441.3	< 1e-07
1457045_at	Galnt13	UDP-N-acetyl-alpha-D-galactosamine:polypeptide N-acetylgalactosaminyltransferase 13	439.1	< 1e-07
1449876_at	Prkg1	protein kinase, cGMP-dependent, type I	413.1	< 1e-07
1426452_a_at	Rsb30	RAB30, member RAS oncogene family	402.9	< 1e-07
1423959_at	Ropn1l	ropporin 1-like	402.6	< 1e-07
1439665_at	Gpr23	G protein-coupled receptor 23	393.4	< 1e-07
1451181_at	2410008J05Rik	RIKEN cDNA 2410008J05 gene	380.3	< 1e-07
1430697_at	Ammecr1	Alport syndrome, mental retardation, midface hypoplasia and elliptocytosis chromosomal region gene 1 homolog (human)	358.5	< 1e-07
1444260_at		Transcribed locus, weakly similar to XP_573711.1 PREDICTED: similar to ORF2	346.3	< 1e-07
1422565_s_at	Nfic	nuclear factor I/C	323.9	< 1e-07

1418876_at	Foxd1	forkhead box D1	310.2	< 1e-07
1435773_at	4930547N16Rik	RIKEN cDNA 4930547N16 gene	291.7	< 1e-07
1460531_at	2900069M18Rik	RIKEN cDNA 2900069M18 gene	291.2	< 1e-07
1428399_a_at	Armc9	armadillo repeat containing 9	266.9	< 1e-07
1446326_at	Col1a2	procollagen, type I, alpha 2	259.7	< 1e-07
1421060_at	Mllt1	myeloid/lymphoid or mixed lineage-leukemia translocation to 1 homolog (Drosophila)	243.3	< 1e-07
1439428_x_at	Gmds	GDP-mannose 4, 6-dehydratase	242.8	< 1e-07
1437835_a_at	0610011L14Rik	RIKEN cDNA 0610011L14 gene	221	< 1e-07
1455859_at	A330021E22Rik	RIKEN cDNA A330021E22 gene	220.2	< 1e-07
1457184_at	4930488L10Rik	FERM domain containing 6 (Frmd6), mRNA	214	< 1e-07
1448458_at	Top2b	topoisomerase (DNA) II beta	204.2	2E-07
1429688_at	Arntl2	aryl hydrocarbon receptor nuclear translocator-like 2	183.3	< 1e-07
1456442_at	Rab3il1	RAB3A interacting protein (rabin3)-like 1	140.3	< 1e-07
1436386_x_at		Transcribed locus	100.1	< 1e-07
1422249_s_at	Zfa /// Zfx	zinc finger protein, autosomal /// zinc finger protein X-linked	97.4	6.5E-05
1457812_at	Trp53bp1	transformation related protein 53 binding protein 1	75.3	< 1e-07
