# Supplementary figures and images for: The molecular signature of therapeutic mesenchymal stem cells exposes the architecture of the hematopoietic stem cell niche synapse
Source: BMC Genomics. 2007 Mar 6;8:65. doi: 10.1186/1471-2164-8-65 (PMC1821333; doi:10.1186/1471-2164-8-65)

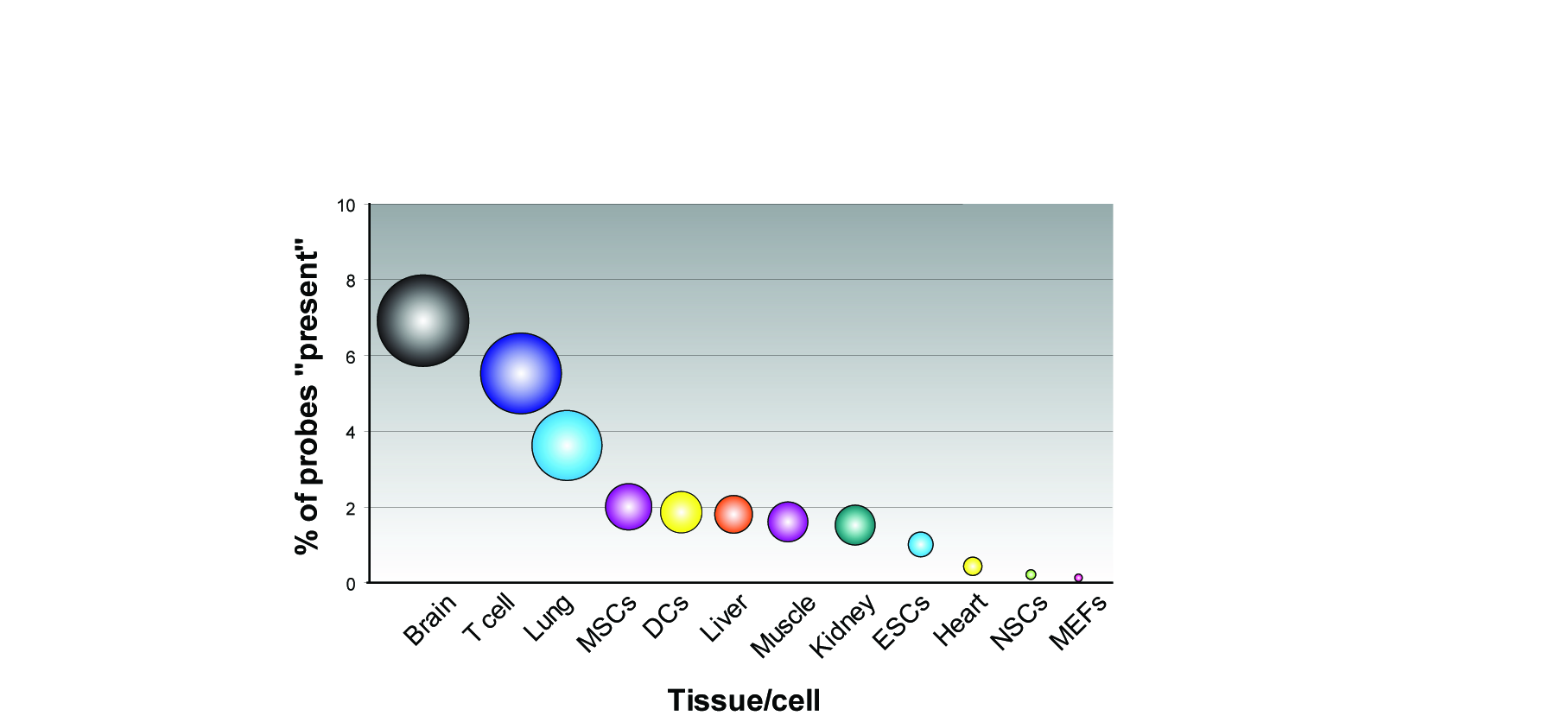

Supplement: Additional file 2 — "Specific" probesets. Samples are represented in circles with different sizes proportional to the absolute amount of probesets detected only in the correspondent tissue or cell type. On Y axis, the proportion of "specific" probesets among all the detected probesets per tissue/cell type is shown. [file 1471-2164-8-65-S2.tiff]
