# Supplementary material for: The molecular signature of therapeutic mesenchymal stem cells exposes the architecture of the hematopoietic stem cell niche synapse
Source: BMC Genomics. 2007 Mar 6;8:65. doi: 10.1186/1471-2164-8-65 (PMC1821333; doi:10.1186/1471-2164-8-65)
Supplement: Additional file 3 — MSC "specific" genes. Genes related to or downstream of the Wnt signaling pathway are highlighted [file 1471-2164-8-65-S3.pdf]

Additional file 3. MSC "specific" genes.

Probe set	Gene symbol	Description	Mean intensity
1451191_at	Crabp2	cellular retinoic acid binding protein II	972.5
1435191_at	Cdsn	corneodesmosin	721.8
1419692_a_at	Ltc4s	leukotriene C4 synthase	585.1
**1416594_at**	**Sfrp1**	**secreted frizzled-related sequence protein 1**	**579.8**
1426127_x_at	Klra18	killer cell lectin-like receptor, subfamily A, member 18	574.0
1456225_x_at	Trib3	tribbles homolog 3 (Drosophila)	522.2
1432057_a_at	Prdm5	PR domain containing 5	483.6
1433653_at	BC029169	cDNA sequence BC029169	482.5
1419671_a_at	Il17rc	interleukin 17 receptor C	457.5
1431834_a_at	Emilin1	elastin microfibril interfacer 1	441.0
1419061_at	Rhod	ras homolog gene family, member D	433.0
**1436207_at**	**Tcf3**	**Transcription factor 3 (Tcf3), mRNA**	**387.9**
1427919_at	Srpx2	sushi-repeat-containing protein, X-linked 2	381.9
1417487_at	Fosl1	fos-like antigen 1	351.0
1452589_at	Ptk7	PTK7 protein tyrosine kinase 7	337.5
**1419416_a_at**	**Rarg**	**retinoic acid receptor, gamma**	**334.9**
1459911_at	D030068L24Rik	RIKEN cDNA D030068L24 gene	331.7
**1427257_at**	**Cspg2**	**chondroitin sulfate proteoglycan 2**	**323.6**
1432092_a_at	Gulp1	GULP, engulfment adaptor PTB domain containing 1	317.8
1450140_a_at	Cdkn2a	cyclin-dependent kinase inhibitor 2A	311.8
1450110_at	Adh7	alcohol dehydrogenase 7 (class IV), mu or sigma polypeptide	307.7
1421952_at	Capn6	calpain 6	306.6
1427764_a_at	Tcfe2a	transcription factor E2a	295.0
1459546_s_at	Enpp1	ectonucleotide pyrophosphatase/phosphodiesterase 1	290.3
1422106_a_at	Spsb2	splA/ryanodine receptor domain and SOCS box containing 2	285.9
1428120_at	Fbxw9	F-box and WD-40 domain protein 9	284.2
1419688_at	Gpc6	glypican 6	283.2
1416516_at	Fscn1	fascin homolog 1, actin bundling protein (Strongylocentrotus) purpuratus)	271.0
1421890_at	St3gal2	ST3 beta-galactoside alpha-2,3-sialyltransferase 2	256.3
1427362_x_at	Hoxc6	homeo box C6	252.3
1426110_a_at	Edg2	endothelial differentiation, lysophosphatidic acid G-protein-coupled receptor, 2	249.8
1421044_at	Mrc2	mannose receptor, C type 2	247.0
1419148_at	Avil	advillin	242.9
1449226_at	Hic1	hypermethylated in cancer 1	236.9
1427361_at	Hoxc6	homeo box C6	230.7
1450325_at	Angpt4	angiopoietin 4	230.7
1424518_at	2310016F22Rik	RIKEN cDNA 2310016F22 gene /// hypothetical protein LOC223672	212.3
1425658_at	Cd109	CD109 antigen	207.9
1431856_a_at	C1qtnf6	C1q and tumor necrosis factor related protein 6	204.6
1447668_x_at	Efemp2	epidermal growth factor-containing fibulin-like extracellular matrix protein 2	204.1
1421227_at	Gzme	granzyme E	202.4
1442595_at		CDNA, clone:Y1G0114E07, strand:unspecified	202.1
1434244_x_at	Htf9c	HpaII tiny fragments locus 9c	199.9

1434795_at	Disp1	dispatched homolog 1 (Drosophila)	199.7
1430521_s_at	Cpne8	copine VIII	198.0
1434241_at	Wdr67	WD repeat domain 67	197.9
1417832_at	Smc1l1	SMC (structural maintenance of chromosomes 1)-like 1 (S. cerevisiae)	196.1
1438603_x_at		Transcribed locus	195.8
1447860_x_at	Cog8	component of oligomeric golgi complex 8	195.5
1456523_at		Adult male urinary bladder cDNA, RIKEN full-length enriched library	194.7
1422647_at	Ring1	ring finger protein 1	194.6
1421001_a_at	Car6	carbonic anhydrase 6	194.4
1427128_at	Ptpn23	protein tyrosine phosphatase, non-receptor type 23	189.0
1449509_at	Serf1	small EDRK-rich factor 1	185.0
1420265_x_at	Cog8	component of oligomeric golgi complex 8	179.8
1439649_at	Adc	arginine decarboxylase	178.3
1436375_at	D530025C17Rik	RIKEN cDNA D530025C17 gene	178.0
1421441_at	Angpt1	angiopoietin 1	177.8
1423378_at	Adam23	a disintegrin and metallopeptidase domain 23	177.1
1460560_at	BC060615	cDNA sequence BC060615	176.1
1419675_at	Ngfb	nerve growth factor, beta	173.8
1448726_at	Snapc2	small nuclear RNA activating complex, polypeptide 2	172.9
1444165_at	E030012M19Rik	RIKEN cDNA E030012M19 gene	171.4
1448432_at	Plcd1	phospholipase C, delta 1	168.0
1449488_at	Pitx1	paired-like homeodomain transcription factor 1	167.9
1425894_at	Mrgprf	MAS-related GPR, member F	165.6
1420414_at	Hoxa11	homeo box A11	164.7
1432295_a_at	2310035K24Rik	RIKEN cDNA 2310035K24 gene	164.5
1421172_at	Adam12	a disintegrin and metallopeptidase domain 12 (meltrin alpha)	164.0
1431753_x_at	2900073H19Rik	RIKEN cDNA 2900073H19 gene	158.9
1430062_at	1600002O04Rik	RIKEN cDNA 1600002O04 gene	156.1
1428241_at	2310035K24Rik	RIKEN cDNA 2310035K24 gene	154.7
1448408_at	Hps1	Hermansky-Pudlak syndrome 1 homolog (human)	153.6
1425126_at	Ncam1	neural cell adhesion molecule 1	153.1
1436517_at	H1fx	H1 histone family, member X	152.3
1439795_at	Gpr64	G protein-coupled receptor 64	149.0
1421309_at	Mgmt	O-6-methylguanine-DNA methyltransferase	148.4
1426973_at	Gpr153	G protein-coupled receptor 153	146.7
1430614_at	4632415K11Rik	RIKEN cDNA 4632415K11 gene	146.4
1447787_x_at	Gja7	gap junction membrane channel protein alpha 7	145.9
1420559_a_at	Shox2	short stature homeobox 2	145.5
1452002_at	BC024969	cDNA sequence BC024969	144.9
1418934_at	Mab21l2	mab-21-like 2 (C. elegans)	144.7
1441070_at			144.5
1438406_at	Scarf2	scavenger receptor class F, member 2	142.7
1455965_at			141.4
1429713_at	Sumf2	PREDICTED: sulfatase modifying factor 2 [Mus musculus], mRNA sequence	140.2
1430354_x_at	Scyl1	SCY1-like 1 (S. cerevisiae)	140.1
1452620_at	Pck2	phosphoenolpyruvate carboxykinase 2 (mitochondrial)	139.7
1431279_s_at	Ttll5	tubulin tyrosine ligase like family 5	138.8
1427688_a_at	Ptprs	protein tyrosine phosphatase, receptor type, S	138.0
1437820_at	Fkhl18	forkhead-like 18 (Drosophila)	136.5
1457827_at	9330196J05Rik	RIKEN cDNA 9330196J05 gene	136.3

1418933_at	Slc1a6	solute carrier family 1 (high affinity aspartate/glutamate transporter), member 6	136.2
1449219_at	Fads3	fatty acid desaturase 3	135.0
1431707_a_at	Pscd3	pleckstrin homology, Sec7 and coiled-coil domains 3	134.3
1460215_at	Rpo1-4	RNA polymerase 1-4	133.8
1455954_x_at	Gpaa1	GPI anchor attachment protein 1	131.7
1451479_a_at	Tmem53	transmembrane protein 53	130.3
1453567_s_at	2810441K11Rik	RIKEN cDNA 2810441K11 gene	130.2
1431015_at	0610039G24Rik	RIKEN cDNA 0610039G24 gene	127.8
1452392_a_at	D11Ertd498e	DNA segment, Chr 11, ERATO Doi 498, expressed	127.1
1442608_at	E030012M19Rik	RIKEN cDNA E030012M19 gene	125.1
1453605_s_at	1810060J02Rik	RIKEN cDNA 1810060J02 gene	124.5
1418919_at	Sgol1	shugoshin-like 1 (S. pombe)	123.1
1450030_at	Dctn4	dynactin 4	121.4
1420565_at	Hoxa1	homeo box A1	121.2
**1417207_at**	**Dvl2**	**dishevelled 2, dsh homolog (Drosophila)**	**119.6**
1418441_at	Col8a1	procollagen, type VIII, alpha 1	119.4
1428863_at	Ankrd39	ankyrin repeat domain 39	112.6
1416599_at	Glis2	GLIS family zinc finger 2	112.5
1420754_at	Ttf1	transcription termination factor 1	112.1
**1426967_at**	**Axin1**	**axin 1**	**112.1**
1423428_at	Ror2	receptor tyrosine kinase-like orphan receptor 2	111.6
1417488_at	Fosl1	fos-like antigen 1	111.4
1431187_s_at	Dlg5	discs, large homolog 5 (Drosophila)	110.9
1435108_at	Arhgap22	Rho GTPase activating protein 22	110.5
1419218_at	Ltap	loop tail associated protein	109.4
1427956_at	Pcgf1	polycomb group ring finger 1	108.4
1449871_at	Tbx18	T-box18	108.0
1435883_at	AW413431	expressed sequence AW413431	107.8
1451779_at	BC027061	cDNA sequence BC027061	107.3
1455962_at	Hhat	hedgehog acyltransferase	105.6
1426452_a_at	Rsb30	RAB30, member RAS oncogene family	105.2
1443636_at	Abcb9	ATP-binding cassette, sub-family B (MDR/TAP), member 9 (Abcb9), mRNA	104.9
1430078_a_at	Ogg1	8-oxoguanine DNA-glycosylase 1	104.9
1452188_at	Maml1	mastermind like 1 (Drosophila)	103.8
1420371_at	Sntb2	syntrophin, basic 2	103.7
1447202_at	1200009F10Rik	RIKEN cDNA 1200009F10 gene	103.0
1425252_a_at	Mad1l1	mitotic arrest deficient 1-like 1	102.5
1417878_at	E2f1	E2F transcription factor 1	101.9
1440617_at	Cpa6	carboxypeptidase A6	101.7
1441412_s_at	Trim45	tripartite motif-containing 45	101.3
1437190_at	Styk1	serine/threonine/tyrosine kinase 1	99.9
1425045_at	Pla2g4b	phospholipase A2, group IVB (cytosolic)	99.1
1453689_at	Fance	Fanconi anemia, complementation group E	98.3
1454924_at	Fut10	fucosyltransferase 10	98.0
1426317_at	Stard6	StAR-related lipid transfer (START) domain containing 6	97.2
1431892_a_at	Plcd3	phospholipase C, delta 3	97.0
1421397_a_at	Lrdd	leucine-rich and death domain containing	96.4
1423483_s_at	Taf1c	TATA box binding protein (Tbp)-associated factor, RNA polymerase I, C	94.9
1436603_at	Tbl2	transducin (beta)-like 2	94.3

1452413_at	C230081A13Rik	RIKEN cDNA C230081A13 gene	94.2
1427316_s_at	AA881470	EST AA881470	93.7
1442819_at	LOC545239	similar to rhomboid-related protein 2	93.6
1439888_at			92.1
1438485_at	E130112L23Rik	RIKEN cDNA E130112L23 gene	91.2
1449169_at	Has2	hyaluronan synthase 2	90.9
1455582_at	C1qtnf1	C1q and tumor necrosis factor related protein 1, mRNA (cDNA clone IMAGE:5052332)	89.5
1418657_at	Znhit4	zinc finger, HIT type 4	89.4
1453214_at	Lrrc15	leucine rich repeat containing 15	89.3
1427699_a_at	Ptpn11	protein tyrosine phosphatase, non-receptor type 11	89.0
1418060_a_at	Mapk7	mitogen activated protein kinase 7	88.3
1435969_at	Btbd12	BTB (POZ) domain containing 12	88.2
1417572_at	Mpg	N-methylpurine-DNA glycosylase	87.7
1451106_at	Rbm21	RNA binding motif protein 21	87.5
1427437_at		PREDICTED: hypothetical protein XP_489268 [Mus musculus], mRNA sequence	86.0
1441968_at	Tspan9	tetraspanin 9	85.8
1425874_at	Hoxc13	homeo box C13	85.7
1418411_at	Fbxl8	F-box and leucine-rich repeat protein 8	84.7
1438139_at	Arhgap28	Rho GTPase activating protein 28	83.9
1441750_x_at	4930447F24Rik	RIKEN cDNA 4930447F24 gene	83.7
1437388_at	Fut10	fucosyltransferase 10	82.4
1459211_at	Gli2	GLI-Kruppel family member GLI2	82.1
1451107_at	Tbc1d22a	TBC1 domain family, member 22a	82.0
1455673_at	AU016977	expressed sequence AU016977	81.7
1453957_a_at	Igf2bp3	insulin-like growth factor 2, binding protein 3	81.0
1441749_at	4930447F24Rik	RIKEN cDNA 4930447F24 gene	80.6
1431680_a_at	Ptprk	protein tyrosine phosphatase, receptor type, K	80.3
1429774_a_at	3830405G04Rik	RIKEN cDNA 3830405G04 gene	79.4
1435132_at	Disp1	dispatched homolog 1 (Drosophila)	78.7
1417548_at	Sart3	squamous cell carcinoma antigen recognized by T-cells 3	77.8
1434289_at	Nsun5	NOL1/NOP2/Sun domain family, member 5	77.5
1424583_at	Farp2	FERM, RhoGEF and pleckstrin domain protein 2	77.4
1423379_at	Nfatc4	nuclear factor of activated T-cells, cytoplasmic, calcineurin-dependent 4	77.0
1426847_at	Sirt4	sirtuin 4 (silent mating type information regulation 2 homolog) 4 (S. cerevisiae)	76.9
1427650_a_at	Runx1	runt related transcription factor 1	76.8
1456559_at	Emx2os	empty spiracles homolog 2 (Drosophila) opposite strand	76.8
1452618_at	Cdk5rap2	CDK5 regulatory subunit associated protein 2	76.8
1441940_x_at	A630065K24Rik	RIKEN cDNA A630065K24 gene	75.3
1421959_s_at	Adcy3	adenylate cyclase 3	74.2
1423468_at	Steap3	STEAP family member 3	73.9
1430701_a_at	5730528L13Rik	RIKEN cDNA 5730528L13 gene	73.6
1451183_at	Myohd1	myosin head domain containing 1	72.8
1423312_at	Tpbg	trophoblast glycoprotein	72.7
1436865_at	Slc26a11	solute carrier family 26, member 11	72.4
1421645_at	9930013L23Rik	RIKEN cDNA 9930013L23 gene	72.3
1452322_a_at	Brwd1	bromodomain and WD repeat domain containing 1	72.1
1449440_at	Lpin3	lipin 3	70.9
1452459_at	Aspm	asp (abnormal spindle)-like, microcephaly associated (Drosophila)	70.9

1423637_at	Galnt4	UDP-N-acetyl-alpha-D-galactosamine:polypeptide N-acetylgalactosaminyltransferase 4	70.9
1420025_s_at	LOC545863	similar to AP2 associated kinase 1	70.5
1458560_at	Aspm	asp (abnormal spindle)-like, microcephaly associated (Drosophila)	70.3
1429517_at	Zfyve20	zinc finger, FYVE domain containing 20	69.5
1451452_a_at	Rgs16	regulator of G-protein signaling 16	69.4
**1448616_at**	**Dvl2**	**dishevelled 2, dsh homolog (Drosophila)**	**69.3**
1456323_at	Pofut1	protein O-fucosyltransferase 1	69.2
1418246_at	Rbm9	RNA binding motif protein 9	68.9
1449549_at	Efnb2	ephrin B2	67.9
1427639_a_at	Nek4	NIMA (never in mitosis gene a)-related expressed kinase 4	67.8
1425876_a_at	MGI:2136405	glucuronyl C5-epimerase	67.6
1422252_a_at	Cdc25c	cell division cycle 25 homolog C (S. cerevisiae)	67.3
1429993_s_at	Speer4b	spermatogenesis associated glutamate (E)-rich protein 4b	67.2
1437857_at	Dpy19l3	dpy-19-like 3 (C. elegans)	66.3
1450050_at	Hira	histone cell cycle regulation defective homolog A (S. cerevisiae)	66.0
1430026_at	Stch	stress 70 protein chaperone, microsome-associated, human homolog	65.7
1425857_at	Fbxw9	F-box and WD-40 domain protein 9, mRNA (cDNA clone IMAGE:5715019)	65.7
1451215_at	1190002C06Rik	RIKEN cDNA 1190002C06 gene	64.7
1439637_at	Kif7	kinesin family member 7	64.5
1452180_at	Phf17	PHD finger protein 17	64.5
1418846_at	Ap4m1	adaptor-related protein complex AP-4, mu 1	64.4
1426795_at	Ptprs	protein tyrosine phosphatase, receptor type, S	64.3
1453302_at	4930429O20Rik	RIKEN cDNA 4930429O20 gene	63.5
1458599_at			63.4
1419686_at	Tsga14	testis specific gene A14	63.2
1422984_at			63.1
1441358_at	Pcdhb16	protocadherin beta 16	63.1
1425824_a_at	Pcsk4	proprotein convertase subtilisin/kexin type 4	61.8
1452687_at	Dus2l	dihydrouridine synthase 2-like (SMM1, S. cerevisiae)	61.5
1425630_at	Sin3b	transcriptional regulator, SIN3B (yeast)	61.4
1421060_at	Mllt1	myeloid/lymphoid or mixed lineage-leukemia translocation to 1 homolog (Drosophila)	61.3
1438579_at	Utp14b	UTP14, U3 small nucleolar ribonucleoprotein, homolog B (yeast)	61.3
1421232_at	Plxna1	plexin A1	60.2
1425994_a_at	Asah2	N-acylsphingosine amidohydrolase 2	60.2
1456950_at	Alms1	Alstrom syndrome 1 homolog (human)	60.2
1442303_at	Anp32a	Acidic (leucine-rich) nuclear phosphoprotein 32 family, member A (Anp32a), mRNA	59.1
1419689_at	Gpc6	glypican 6	58.8
1421512_at	Cep2	centrosomal protein 2	58.0
1422741_a_at	Bbx	bobby sox homolog (Drosophila)	57.9
1447994_at	1700026B20Rik	RIKEN cDNA 1700026B20 gene	57.9
1460080_at	AI645535	expressed sequence AI645535	57.8
1442203_at	LOC552880	hypothetical LOC552880	57.2
1458660_at			57.1
1436063_at	Loxl1	lysyl oxidase-like 1	56.7
1427093_at	Zfp707	zinc finger protein 707	56.3
1436414_at	AW822216	expressed sequence AW822216	56.1
1438490_at	Slc39a14	solute carrier family 39 (zinc transporter), member 14	55.8
1431315_at	Hyls1	hydrolethalus syndrome 1	55.7

1444016_at	C430014K04Rik	RIKEN cDNA C430014K04 gene	55.4
1434409_at	AW060994	expressed sequence AW060994	55.2
1455786_at	2610036F08Rik	RIKEN cDNA 2610036F08 gene, mRNA (cDNA clone MGC:29393 IMAGE:5066012)	55.0
1429484_at	1110002L01Rik	RIKEN cDNA 1110002L01 gene	54.6
1428983_at	Scx	scleraxis	53.9
1437835_a_at	0610011L14Rik	RIKEN cDNA 0610011L14 gene	53.8
1426040_a_at	Odf2	outer dense fiber of sperm tails 2	53.8
1431351_at	Pvt1	plasmacytoma variant translocation 1	53.7
1445451_at			53.6
1425118_at	Spire2	spire homolog 2 (Drosophila)	53.5
1420097_at	D13Ertd787e	DNA segment, Chr 13, ERATO Doi 787, expressed	53.4
1454369_a_at	Nfatc4	nuclear factor of activated T-cells, cytoplasmic, calcineurin-dependent 4	52.8
1452978_at	2900055D14Rik	RIKEN cDNA 2900055D14 gene	52.8
1444704_at	D5Ertd606e	Septin 11, mRNA (cDNA clone MGC:28420 IMAGE:4037515)	52.8
1447890_at	Rcn1	reticulocalbin 1	52.5
1455881_at	Ier5l	immediate early response 5-like	52.4
1430427_a_at	Pcdh18	protocadherin 18	52.3
1429895_at	2310010G23Rik	RIKEN cDNA 2310010G23 gene	52.3
1431070_a_at	9130404D08Rik	RIKEN cDNA 9130404D08 gene	51.8
1440402_at	4831426I19Rik	RIKEN cDNA 4831426I19 gene	51.7
1435713_at	Mettl2	methyltransferase like 2	51.6
1439465_x_at	2310016E02Rik	RIKEN cDNA 2310016E02 gene /// RIKEN cDNA 9430057O19 gene	51.0
1436442_at	Ptpn14	protein tyrosine phosphatase, non-receptor type 14	49.9
1431297_a_at	4933436C20Rik	RIKEN cDNA 4933436C20 gene	49.8
1427015_at	LOC380969	similar to KIAA1602 protein	49.7
1417198_at	D8Ertd594e	DNA segment, Chr 8, ERATO Doi 594, expressed	49.5
1449358_at	D6Mm5e	DNA segment, Chr 6, Miriam Meisler 5, expressed	48.9
1438350_at	Gpr64	G protein-coupled receptor 64	48.4
1429688_at	Arntl2	aryl hydrocarbon receptor nuclear translocator-like 2	47.8
1437330_at	Lrrk1	leucine-rich repeat kinase 1	47.7
1452609_at	1190005I06Rik	RIKEN cDNA 1190005I06 gene	47.3
1454558_at	5430416B10Rik	RIKEN cDNA 5430416B10 gene	47.1
1435946_at	D5Ertd135e	DNA segment, Chr 5, ERATO Doi 135, expressed	47.1
1421296_at	Tnfrsf10b	tumor necrosis factor receptor superfamily, member 10b	46.5
1456647_a_at	Eya4	eyes absent 4 homolog (Drosophila)	46.3
1421171_at	Adam12	a disintegrin and metallopeptidase domain 12 (meltrin alpha)	46.1
1420391_at	Pard3	par-3 (partitioning defective 3) homolog (C. elegans)	46.0
1419937_at	AA536749	expressed sequence AA536749	45.9
1443394_at	1700006J14Rik	RIKEN cDNA 1700006J14 gene (1700006J14Rik), mRNA	45.7
1455049_at	Igsf3	immunoglobulin superfamily, member 3	45.6
1439885_at	Hoxc5	homeo box C5	45.0
1438293_at	A930031F18Rik	RIKEN cDNA A930031F18 gene	44.9
1437655_at	D430020J02Rik	RIKEN cDNA D430020J02 gene	44.8
1417117_at	Cstf1	cleavage stimulation factor, 3' pre-RNA, subunit 1	44.8
1432284_at	Mipol1	mirror-image polydactyly gene 1 homolog (human)	43.9
1420848_at	Sufu	suppressor of fused homolog (Drosophila)	43.6
1425056_s_at	Saps2	SAPS domain family, member 2	43.6
1444105_at	Acta2	actin, alpha 2, smooth muscle, aorta	43.5
1417304_at	Chrd	chordin	43.3
1456937_at	Cdh26	cadherin-like 26	42.8

1458050_at			42.5
1439028_at	1810045K17Rik	Ubiquitin-fold modifier 1 (Ufm1), mRNA	42.4
1450687_at	Igf2bp3	insulin-like growth factor 2, binding protein 3	42.4
1429611_at	1700034E13Rik	RIKEN cDNA 1700034E13 gene	42.3
1422039_at	Tnfrsf22	tumor necrosis factor receptor superfamily, member 22	41.9
1441125_at	2900045N06Rik	RIKEN cDNA 2900045N06 gene	41.8
1431270_a_at	1100001A21Rik	RIKEN cDNA 1100001A21 gene	41.4
1430247_at	Daam2	dishevelled associated activator of morphogenesis 2	40.7
1427617_at	Fut10	fucosyltransferase 10	40.6
1424561_at	Ece2	endothelin converting enzyme 2	40.2
1443065_at	4930418P06Rik	RIKEN cDNA 4930418P06 gene (4930418P06Rik), mRNA	40.0
1449558_at	F8	coagulation factor VIII	39.3
1432448_at	2600006K01Rik	RIKEN cDNA 2600006K01 gene	39.3
1450307_x_at	H2afy2	H2A histone family, member Y2	39.0
1423761_at	5630401D24Rik	RIKEN cDNA 5630401D24 gene	38.7
1431409_at	C030015A19Rik	RIKEN cDNA C030015A19 gene	38.7
1453708_a_at	Gsto2	glutathione S-transferase omega 2	38.6
1456329_at	A230098A12Rik	RIKEN cDNA A230098A12 gene	38.5
1441520_at	Aspm	asp (abnormal spindle)-like, microcephaly associated (Drosophila)	37.9
1431088_at	Foxp4	forkhead box P4	37.7
1419679_at	Lats2	large tumor suppressor 2	37.5
1441367_a_at		Transcribed locus	37.0
1437903_at	Lox	lysyl oxidase	37.0
1427659_at	Smarcd1	SWI/SNF related, matrix associated, actin dependent regulator of chromatin, subfamily d, member 1	36.7
1427454_at	Hoxc6	homeo box C6	36.6
1438479_at	BC038178	cDNA sequence BC038178	36.1
1449215_at	Slc22a21	solute carrier family 22 (organic cation transporter), member 21	35.2
1438488_at	Esd	esterase D/formylglutathione hydrolase	35.1
1431162_a_at	Enah	enabled homolog (Drosophila)	34.9
1457280_at			33.9
1449392_at	Hsd17b1	hydroxysteroid (17-beta) dehydrogenase 1	33.7
1460744_at	2810002I04Rik	RIKEN cDNA 2810002I04 gene, mRNA (cDNA clone MGC:54815 IMAGE:6305959)	33.2
1443379_at	A430033K04Rik	RIKEN cDNA A430033K04 gene	33.1
1426695_at	9030624J02Rik	RIKEN cDNA 9030624J02 gene	32.9
1452503_a_at	Brwd1	bromodomain and WD repeat domain containing 1	32.5
1454460_at	5730433N10Rik	RIKEN cDNA 5730433N10 gene	32.3
1457847_at			32.0
1442781_at			31.8
1420443_at	Pcdhb19	protocadherin beta 19	31.5
1447973_at	Trip11	thyroid hormone receptor interactor 11	31.4
1459203_at	Ipo8	importin 8	31.2
1423056_at	Nsg1	neuron specific gene family member 1	31.1
1453754_at	4930429A08Rik	RIKEN cDNA 4930429A08 gene	30.6
1431353_at	C330050A14Rik	RIKEN cDNA C330050A14 gene	30.4
1459804_at		Adult male aorta and vein cDNA, RIKEN full-length enriched library, clone:A530003I17	29.8
1460483_at	2610034E01Rik	RIKEN cDNA 2610034E01 gene	29.7
1446894_at	Arnt2	Aryl hydrocarbon receptor nuclear translocator 2 (Arnt2), mRNA	29.7
1442383_at		CDNA clone MGC:107704 IMAGE:6831822	29.3

1420516_at	a	nonagouti	28.4
1431417_at	Jam2	junction adhesion molecule 2	28.3
1442120_at			28.3
1443346_at	2700007P21Rik	RIKEN cDNA 2700007P21 gene	28.0
1453633_a_at	Rnf41	ring finger protein 41	27.4
1431017_at	Rcbtb2	regulator of chromosome condensation (RCC1) and BTB (POZ) domain containing protein 2	27.3
1437935_at	4930486G11Rik	RIKEN cDNA 4930486G11 gene	27.2
1429405_at	2010317E24Rik	RIKEN cDNA 2010317E24 gene	27.0
1434186_at	Gpr23	G protein-coupled receptor 23	26.6
1416154_at	Srp54	signal recognition particle 54	26.5
1442314_at	Nup188	nucleoporin 188	26.5
1439116_at			26.4
1452538_at	Igh-V3609N	Anti-brucella monoclonal antibody A76 12G12 immunoglobulin heavy chain (IGH gene)	26.2
1443716_at	LOC545331	hypothetical protein LOC545331	26.1
1422148_at	Matn3	matrilin 3	25.9
1446804_at			25.9
1422726_x_at	Speer4a	spermatogenesis associated glutamate (E)-rich protein 4a	25.9
1440282_at	Tulp4 /// 2210038L17Rik	tubby like protein 4 /// RIKEN cDNA 2210038L17 gene	25.4
1446498_at	E230031K19	hypothetical protein E230031K19	24.9
1431330_at	3110002H16Rik	RIKEN cDNA 3110002H16 gene	24.8
1425775_at	2610036F08Rik	RIKEN cDNA 2610036F08 gene	24.7
1449683_x_at	2410129E14Rik	RIKEN cDNA 2410129E14 gene	24.4
1441339_at	Chd9	chromodomain helicase DNA binding protein 9	23.7
1440875_a_at	Rsad1	radical S-adenosyl methionine domain containing 1	23.3
1445445_s_at	Ptger1	prostaglandin E receptor 1 (subtype EP1)	23.3
1430847_a_at	Crem	cAMP responsive element modulator	22.8
1454013_at	1810062O18Rik	RIKEN cDNA 1810062O18 gene	22.3
1460065_at	AA763515	expressed sequence AA763515	22.2
1443792_at	Tsga14	testis specific gene A14	21.9
1438804_at	Sept10	septin 10	20.9
1425765_at	Rrn3	RRN3 RNA polymerase I transcription factor homolog (yeast), mRNA	20.5
1431669_at	4930583I09Rik	RIKEN cDNA 4930583I09 gene	20.4
1456554_at	7530414M10Rik	RIKEN cDNA 7530414M10 gene	20.1
1456820_at	Fbxl19	F-box and leucine-rich repeat protein 19, mRNA (cDNA clone MGC:69570 IMAGE:6401846)	19.3
1427499_at			18.8
1421979_at	Phex	phosphate regulating gene with homologies to endopeptidases on the X chromosome	18.7
1455416_at	C130021I20	hypothetical protein C130021I20	18.6
1440741_at	Htr1d	5-hydroxytryptamine (serotonin) receptor 1D	18.5
1444156_at	9230112E08Rik	RIKEN cDNA 9230112E08 gene	17.5
1436922_at			16.7
1442192_at	Tyms	thymidylate synthase	16.4
1442352_at	9430091N11Rik	RIKEN cDNA 9430091N11 gene	16.4
1445907_at			16.3
1457326_at			15.9
1457010_at	3010001F23Rik	RIKEN cDNA 3010001F23 gene	12.5
