# Supplementary material for: The molecular signature of therapeutic mesenchymal stem cells exposes the architecture of the hematopoietic stem cell niche synapse
Source: BMC Genomics. 2007 Mar 6;8:65. doi: 10.1186/1471-2164-8-65 (PMC1821333; doi:10.1186/1471-2164-8-65)
Supplement: Additional file 4 — Candidate molecules for MSC biological activities. A compilation of molecules potentially regulating the activity of MSCs. [file 1471-2164-8-65-S4.pdf]

Additional file 4. Candidate molecules for MSC biological activities.

**Candidate gene**	**Described in MSCs?**	**Stem cell niche?**	**Inhibition of proliferation?**
**Vcam1**	Yes	Yes	No
**Cxcl12**	Yes	Yes	No
**Angpt1**	No	Yes	Yes
**Spp1**	Yes	Yes	Yes
**Thbs1**	Yes	Yes	Yes
**Thbs2**	Yes	Yes	Yes
**Fgf7**	Yes	No	No
**Sema3a**	Yes	No	Yes
**Serpinf1**	Yes	Yes	Yes
**Igfbp-4**	Yes	Yes	Yes
**Fn1**	Yes	Yes	Yes
**Sparc**	Yes	No	Yes
**Wnt5b**	Yes	No	No
**Wisp1**	Yes	No	No
**Sfrp1**	Yes	Yes	Yes
**Sfrp2**	Yes	Yes	Yes
**Dkk3**	Yes	Yes	Yes
**Lgals1**	Yes	Yes	Yes
**Tgfb2**	Yes	Yes	Yes
**Cyr61**	Yes	No	No
