# Supplementary material for: The molecular signature of therapeutic mesenchymal stem cells exposes the architecture of the hematopoietic stem cell niche synapse
Source: BMC Genomics. 2007 Mar 6;8:65. doi: 10.1186/1471-2164-8-65 (PMC1821333; doi:10.1186/1471-2164-8-65)
Supplement: Additional file 5 — Expression datasets used in this article. Description of microarray experiments used in this article. For each experiment, the laboratory of origin, the tissue and the Affymetrix platform are indicated. [file 1471-2164-8-65-S5.pdf]

Additional file 5. Expression datasets used in this article

Array ID	Tissue	Lab	Platform
GSM113088	mesenchymal stem cells	Oksenberg	423 2.0
GSM113089	mesenchymal stem cells	Oksenberg	424 2.0
GSM113091	mesenchymal stem cells	Oksenberg	425 2.0
GSM113092	mesenchymal stem cells	Oksenberg	426 2.0
GSM113093	mesenchymal stem cells	Oksenberg	427 2.0
GSM113094	mesenchymal stem cells	Oksenberg	428 2.0
GSM113095	mesenchymal stem cells	Oksenberg	429 2.0
GSM72507	brain	Johnson	430 2.0
GSM73018	brain	Johnson	430 2.0
GSM73019	brain	Johnson	430 2.0
GSM73001	brain	Johnson	430 2.0
GSM73014	brain	Johnson	430 2.0
GSM73015	brain	Johnson	430 2.0
GSM73000	brain	Johnson	430 2.0
GSM73340	brain	Johnson	430 2.0
GSM73341	brain	Johnson	430 2.0
GSM73002	brain	Johnson	430 2.0
GSM73016	brain	Johnson	430 2.0
GSM73017	brain	Johnson	430 2.0
GSM35884	brain	Wilson	430 2.0
GSM35885	brain	Wilson	430 2.0
GSM35886	brain	Wilson	430 2.0
GSM35887	brain	Wilson	430 2.0
GSM35888	brain	Wilson	430 2.0
GSM35889	brain	Wilson	430 2.0
GSM35890	brain	Wilson	430 2.0
GSM35891	brain	Wilson	430 2.0
GSM35892	brain	Wilson	430 2.0
GSM35893	brain	Wilson	430 2.0
GSM35894	brain	Wilson	430 2.0
GSM35895	brain	Wilson	430 2.0
GSM35896	brain	Wilson	430 2.0
GSM35897	brain	Wilson	430 2.0
GSM35898	brain	Wilson	430 2.0
GSM92518	brain	Zhang	430 2.0
GSM92519	brain	Zhang	430 2.0
GSM92520	brain	Zhang	430 2.0
GSM92521	brain	Zhang	430 2.0
GSM92522	brain	Zhang	430 2.0
GSM92523	brain	Zhang	430 2.0
GSM92512	brain	Sikes	430 2.0
GSM92513	brain	Sikes	430 2.0
GSM92514	brain	Sikes	430 2.0
GSM92515	brain	Sikes	430 2.0
GSM92516	brain	Sikes	430 2.0
GSM92517	brain	Sikes	430 2.0
GSM92435	brain	Palme	430 2.0
GSM92436	brain	Palme	430 2.0
GSM92437	brain	Palme	430 2.0
GSM92438	brain	Palme	430 2.0

GSM92439	brain	Palme	430 2.0
GSM92440	brain	Palme	430 2.0
GSM92441	brain	Palme	430 2.0
GSM92442	brain	Palme	430 2.0
GSM92443	brain	Palme	430 2.0
GSM92444	brain	Palme	430 2.0
GSM92445	brain	Palme	430 2.0
GSM92446	brain	Palme	430 2.0
GSM92447	brain	Palme	430 2.0
GSM92448	brain	Palme	430 2.0
GSM92449	brain	Palme	430 2.0
GSM92450	brain	Palme	430 2.0
GSM92451	brain	Palme	430 2.0
GSM92452	brain	Palme	430 2.0
GSM92409	brain	Palme	430 2.0
GSM92410	brain	Palme	430 2.0
GSM92411	brain	Palme	430 2.0
GSM92412	brain	Palme	430 2.0
GSM92413	brain	Palme	430 2.0
GSM92414	brain	Palme	430 2.0
GSM92415	brain	Palme	430 2.0
GSM92416	brain	Palme	430 2.0
GSM92417	brain	Palme	430 2.0
GSM92418	brain	Palme	430 2.0
GSM92419	brain	Palme	430 2.0
GSM92420	brain	Palme	430 2.0
GSM92421	brain	Palme	430 2.0
GSM92422	brain	Palme	430 2.0
GSM92423	brain	Palme	430 2.0
GSM92424	brain	Palme	430 2.0
GSM92425	brain	Palme	430 2.0
GSM92426	brain	Palme	430 2.0
GSM67177	brain	Sikela	430A
GSM67181	brain	Sikela	430A
GSM67184	brain	Sikela	430A
GSM67185	brain	Sikela	430A
GSM67187	brain	Sikela	430B
GSM67192	brain	Sikela	430B
GSM67193	brain	Sikela	430B
GSM38974	brain	Rubenstein	430 2.0
GSM38975	brain	Rubenstein	430 2.0
GSM38976	brain	Rubenstein	430 2.0
GSM38977	brain	Rubenstein	430 2.0
GSM38978	brain	Rubenstein	430 2.0
GSM38979	brain	Rubenstein	430 2.0
GSM38980	brain	Rubenstein	430 2.0
GSM38981	brain	Rubenstein	430 2.0
GSM40050	dendritic cells	Medzhitov	430 2.0
GSM40051	dendritic cells	Medzhitov	430 2.0
GSM40053	dendritic cells	Medzhitov	430 2.0
GSM40054	dendritic cells	Medzhitov	430 2.0
GSM40055	dendritic cells	Medzhitov	430 2.0

GSM40056	dendritic cells	Medzhitov	430 2.0
GSM67326	embryonic fibroblasts	Brindle	430 2.0
GSM67327	embryonic fibroblasts	Brindle	430 2.0
GSM67328	embryonic fibroblasts	Brindle	430 2.0
GSM67329	embryonic fibroblasts	Brindle	430 2.0
GSM67330	embryonic fibroblasts	Brindle	430 2.0
GSM67331	embryonic fibroblasts	Brindle	430 2.0
GSM67332	embryonic fibroblasts	Brindle	430 2.0
GSM67333	embryonic fibroblasts	Brindle	430 2.0
GSM67334	embryonic fibroblasts	Brindle	430 2.0
GSM67335	embryonic fibroblasts	Brindle	430 2.0
GSM67336	embryonic fibroblasts	Brindle	430 2.0
GSM67337	embryonic fibroblasts	Brindle	430 2.0
GSM67338	embryonic fibroblasts	Brindle	430 2.0
GSM67339	embryonic fibroblasts	Brindle	430 2.0
GSM67340	embryonic fibroblasts	Brindle	430 2.0
GSM67341	embryonic fibroblasts	Brindle	430 2.0
GSM67342	embryonic fibroblasts	Brindle	430 2.0
GSM67343	embryonic fibroblasts	Brindle	430 2.0
GSM67344	embryonic fibroblasts	Brindle	430 2.0
GSM67345	embryonic fibroblasts	Brindle	430 2.0
GSM67346	embryonic fibroblasts	Brindle	430 2.0
GSM67347	embryonic fibroblasts	Brindle	430 2.0
GSM67348	embryonic fibroblasts	Brindle	430 2.0
GSM67349	embryonic fibroblasts	Brindle	430 2.0
GSM67350	embryonic fibroblasts	Brindle	430 2.0
GSM67351	embryonic fibroblasts	Brindle	430 2.0
GSM67352	embryonic fibroblasts	Brindle	430 2.0
GSM67353	embryonic fibroblasts	Brindle	430 2.0
GSM67322	embryonic fibroblasts	Brindle	430A
GSM67323	embryonic fibroblasts	Brindle	430A
GSM67324	embryonic fibroblasts	Brindle	430A
GSM67325	embryonic fibroblasts	Brindle	430A
GSM72918	embryonic fibroblasts	Rudnicki	430A
GSM72920	embryonic fibroblasts	Rudnicki	430A
GSM72922	embryonic fibroblasts	Rudnicki	430A
GSM72919	embryonic fibroblasts	Rudnicki	430B
GSM72921	embryonic fibroblasts	Rudnicki	430B
GSM72923	embryonic fibroblasts	Rudnicki	430B
GSM72870	embryonic fibroblasts	Rudnicki	430A
GSM72872	embryonic fibroblasts	Rudnicki	430A
GSM72874	embryonic fibroblasts	Rudnicki	430A
GSM72871	embryonic fibroblasts	Rudnicki	430B
GSM72873	embryonic fibroblasts	Rudnicki	430B
GSM72875	embryonic fibroblasts	Rudnicki	430B
GSM40010	embryonic stem cells	Speed	430 2.0
GSM40011	embryonic stem cells	Speed	430 2.0
GSM40012	embryonic stem cells	Speed	430 2.0
GSM40013	embryonic stem cells	Speed	430 2.0
GSM94856	embryonic stem cells	Ng	430 2.0
GSM94857	embryonic stem cells	Ng	430 2.0
GSM94858	embryonic stem cells	Ng	430 2.0

GSM94859	embryonic stem cells	Ng	430 2.0
GSM94860	embryonic stem cells	Ng	430 2.0
GSM94861	embryonic stem cells	Ng	430 2.0
GSM94862	embryonic stem cells	Ng	430 2.0
GSM94863	embryonic stem cells	Ng	430 2.0
GSM94864	embryonic stem cells	Ng	430 2.0
GSM94865	embryonic stem cells	Ng	430 2.0
GSM94866	embryonic stem cells	Ng	430 2.0
GSM94867	embryonic stem cells	Ng	430 2.0
GSM94868	embryonic stem cells	Ng	430 2.0
GSM94869	embryonic stem cells	Ng	430 2.0
GSM93570	embryonic stem cells	Gertsenstein	430A
GSM93572	embryonic stem cells	Gertsenstein	430A
GSM93574	embryonic stem cells	Gertsenstein	430A
GSM93576	embryonic stem cells	Gertsenstein	430A
GSM93578	embryonic stem cells	Gertsenstein	430A
GSM93580	embryonic stem cells	Gertsenstein	430A
GSM93582	embryonic stem cells	Gertsenstein	430A
GSM93584	embryonic stem cells	Gertsenstein	430A
GSM93586	embryonic stem cells	Gertsenstein	430A
GSM93571	embryonic stem cells	Gertsenstein	430B
GSM93573	embryonic stem cells	Gertsenstein	430B
GSM93575	embryonic stem cells	Gertsenstein	430B
GSM93577	embryonic stem cells	Gertsenstein	430B
GSM93579	embryonic stem cells	Gertsenstein	430B
GSM93581	embryonic stem cells	Gertsenstein	430B
GSM93583	embryonic stem cells	Gertsenstein	430B
GSM93585	embryonic stem cells	Gertsenstein	430B
GSM93587	embryonic stem cells	Gertsenstein	430B
GSM86589	embryonic stem cells	Piret	430A
GSM86591	embryonic stem cells	Piret	430A
GSM86593	embryonic stem cells	Piret	430A
GSM86590	embryonic stem cells	Piret	430B
GSM86592	embryonic stem cells	Piret	430B
GSM86594	embryonic stem cells	Piret	430B
GSM72616	embryonic stem cells	Rudnicki	430A
GSM72618	embryonic stem cells	Rudnicki	430A
GSM72621	embryonic stem cells	Rudnicki	430A
GSM72622	embryonic stem cells	Rudnicki	430A
GSM72624	embryonic stem cells	Rudnicki	430A
GSM72626	embryonic stem cells	Rudnicki	430A
GSM72617	embryonic stem cells	Rudnicki	430B
GSM72619	embryonic stem cells	Rudnicki	430B
GSM72620	embryonic stem cells	Rudnicki	430B
GSM72623	embryonic stem cells	Rudnicki	430B
GSM72625	embryonic stem cells	Rudnicki	430B
GSM72627	embryonic stem cells	Rudnicki	430B
GSM64922	embryonic stem cells	Rudnicki	430A
GSM64924	embryonic stem cells	Rudnicki	430A
GSM64926	embryonic stem cells	Rudnicki	430A
GSM64923	embryonic stem cells	Rudnicki	430B
GSM64925	embryonic stem cells	Rudnicki	430B

GSM64927	embryonic stem cells	Rudnicki	430B
GSM80525	heart	Wang	430 2.0
GSM80526	heart	Wang	430 2.0
GSM80527	heart	Wang	430 2.0
GSM80528	heart	Wang	430 2.0
GSM80529	heart	Wang	430 2.0
GSM80530	heart	Wang	430 2.0
GSM80531	heart	Wang	430 2.0
GSM80532	heart	Wang	430 2.0
GSM80533	heart	Wang	430 2.0
GSM80534	heart	Wang	430 2.0
GSM80535	heart	Wang	430 2.0
GSM80536	heart	Wang	430 2.0
GSM80537	heart	Wang	430 2.0
GSM80538	heart	Wang	430 2.0
GSM80539	heart	Wang	430 2.0
GSM80540	heart	Wang	430 2.0
GSM80541	heart	Wang	430 2.0
GSM80542	heart	Wang	430 2.0
GSM77688	heart	Turchin	430 2.0
GSM77689	heart	Turchin	430 2.0
GSM77690	heart	Turchin	430 2.0
GSM77691	heart	Turchin	430 2.0
GSM77692	heart	Turchin	430 2.0
GSM77693	heart	Turchin	430 2.0
GSM77694	heart	Turchin	430 2.0
GSM77695	heart	Turchin	430 2.0
GSM77696	heart	Turchin	430 2.0
GSM77697	heart	Turchin	430 2.0
GSM77698	heart	Turchin	430 2.0
GSM77699	heart	Turchin	430 2.0
GSM77700	heart	Turchin	430 2.0
GSM77701	heart	Turchin	430 2.0
GSM77702	heart	Turchin	430 2.0
GSM40959	heart	Shirasawa	430 2.0
GSM40960	heart	Shirasawa	430 2.0
GSM40957	heart	Shirasawa	430 2.0
GSM40958	heart	Shirasawa	430 2.0
GSM67362	heart	Portman	430 2.0
GSM67363	heart	Portman	430 2.0
GSM67364	heart	Portman	430 2.0
GSM67365	heart	Portman	430 2.0
GSM67366	heart	Portman	430 2.0
GSM67367	heart	Portman	430 2.0
GSM67368	heart	Portman	430 2.0
GSM67369	heart	Portman	430 2.0
GSM67370	heart	Portman	430 2.0
GSM67371	heart	Portman	430 2.0
GSM67372	heart	Portman	430 2.0
GSM67373	heart	Portman	430 2.0
GSM67374	heart	Portman	430 2.0
GSM67375	heart	Portman	430 2.0

GSM67376	heart	Portman	430 2.0
GSM67377	heart	Portman	430 2.0
GSM67378	heart	Portman	430 2.0
GSM67379	heart	Portman	430 2.0
GSM25150	heart	Harvard	430 2.0
GSM25151	heart	Harvard	430 2.0
GSM25152	heart	Harvard	430 2.0
GSM25153	heart	Harvard	430 2.0
GSM25154	heart	Harvard	430 2.0
GSM25155	heart	Harvard	430 2.0
GSM25156	heart	Harvard	430 2.0
GSM25157	heart	Harvard	430 2.0
GSM25158	heart	Harvard	430 2.0
GSM25159	heart	Harvard	430 2.0
GSM25160	heart	Harvard	430 2.0
GSM25161	heart	Harvard	430 2.0
GSM25162	heart	Harvard	430 2.0
GSM25163	heart	Harvard	430 2.0
GSM25164	heart	Harvard	430 2.0
GSM25165	heart	Harvard	430 2.0
GSM25166	heart	Harvard	430 2.0
GSM25167	heart	Harvard	430 2.0
GSM72978	hematopoietic stem cells	Rudnicki	430A
GSM72980	hematopoietic stem cells	Rudnicki	430A
GSM72979	hematopoietic stem cells	Rudnicki	430B
GSM72981	hematopoietic stem cells	Rudnicki	430B
GSM36470	kidney	Wangemann	430 2.0
GSM36471	kidney	Wangemann	430 2.0
GSM36472	kidney	Wangemann	430 2.0
GSM24066	kidney	Wangemann	430 2.0
GSM24067	kidney	Wangemann	430 2.0
GSM24065	kidney	Wangemann	430 2.0
GSM24068	kidney	Wangemann	430 2.0
GSM24069	kidney	Wangemann	430 2.0
GSM24070	kidney	Wangemann	430 2.0
GSM24071	kidney	Wangemann	430 2.0
GSM24072	kidney	Wangemann	430 2.0
GSM24073	kidney	Wangemann	430 2.0
GSM24074	kidney	Wangemann	430 2.0
GSM96563	kidney	Behringer	430 2.0
GSM96564	kidney	Behringer	430 2.0
GSM96565	kidney	Behringer	430 2.0
GSM96566	kidney	Behringer	430 2.0
GSM96567	kidney	Behringer	430 2.0
GSM96568	kidney	Behringer	430 2.0
GSM96569	kidney	Behringer	430 2.0
GSM96570	kidney	Behringer	430 2.0
GSM88872	liver	Attie	430 2.0
GSM88873	liver	Attie	430 2.0
GSM88874	liver	Attie	430 2.0
GSM88875	liver	Attie	430 2.0
GSM88876	liver	Attie	430 2.0

GSM88877	liver	Attie	430 2.0
GSM88878	liver	Attie	430 2.0
GSM88879	liver	Attie	430 2.0
GSM88880	liver	Attie	430 2.0
GSM88881	liver	Attie	430 2.0
GSM88882	liver	Attie	430 2.0
GSM88883	liver	Attie	430 2.0
GSM88884	liver	Attie	430 2.0
GSM88885	liver	Attie	430 2.0
GSM88886	liver	Attie	430 2.0
GSM88887	liver	Attie	430 2.0
GSM88888	liver	Attie	430 2.0
GSM88889	liver	Attie	430 2.0
GSM88890	liver	Attie	430 2.0
GSM88891	liver	Attie	430 2.0
GSM87833	liver	McCabe	430 2.0
GSM87834	liver	McCabe	430 2.0
GSM87835	liver	McCabe	430 2.0
GSM87836	liver	McCabe	430 2.0
GSM87837	liver	McCabe	430 2.0
GSM87838	liver	McCabe	430 2.0
GSM87839	liver	McCabe	430 2.0
GSM87840	liver	McCabe	430 2.0
GSM77216	liver	Rockefeller	430 2.0
GSM77217	liver	Rockefeller	430 2.0
GSM75026	liver	Attie	430A
GSM75027	liver	Attie	430A
GSM75028	liver	Attie	430A
GSM75029	liver	Attie	430A
GSM75030	liver	Attie	430A
GSM75031	liver	Attie	430A
GSM75032	liver	Attie	430A
GSM75033	liver	Attie	430A
GSM75034	liver	Attie	430A
GSM75035	liver	Attie	430A
GSM75036	liver	Attie	430A
GSM75037	liver	Attie	430A
GSM75038	liver	Attie	430A
GSM75039	liver	Attie	430A
GSM75040	liver	Attie	430A
GSM75041	liver	Attie	430A
GSM75042	liver	Attie	430A
GSM75043	liver	Attie	430A
GSM68907	liver	DeFord	430 2.0
GSM68908	liver	DeFord	430 2.0
GSM68909	liver	DeFord	430 2.0
GSM68910	liver	DeFord	430 2.0
GSM68911	liver	DeFord	430 2.0
GSM68912	liver	DeFord	430 2.0
GSM68913	liver	DeFord	430 2.0
GSM68914	liver	DeFord	430 2.0
GSM68915	liver	DeFord	430 2.0

GSM68916	liver	DeFord	430 2.0
GSM68917	liver	DeFord	430 2.0
GSM68918	liver	DeFord	430 2.0
GSM68919	liver	DeFord	430 2.0
GSM68920	liver	DeFord	430 2.0
GSM68921	liver	DeFord	430 2.0
GSM68922	liver	DeFord	430 2.0
GSM68923	liver	DeFord	430 2.0
GSM68924	liver	DeFord	430 2.0
GSM69792	liver	Duncan	430 2.0
GSM69793	liver	Duncan	430 2.0
GSM69794	liver	Duncan	430 2.0
GSM69795	liver	Duncan	430 2.0
GSM69796	liver	Duncan	430 2.0
GSM69797	liver	Duncan	430 2.0
GSM29715	lung	Johns Hopkins	430 2.0
GSM29716	lung	Johns Hopkins	430 2.0
GSM29717	lung	Johns Hopkins	430 2.0
GSM29712	lung	Johns Hopkins	430 2.0
GSM29713	lung	Johns Hopkins	430 2.0
GSM29714	lung	Johns Hopkins	430 2.0
GSM29709	lung	Johns Hopkins	430 2.0
GSM29710	lung	Johns Hopkins	430 2.0
GSM29711	lung	Johns Hopkins	430 2.0
GSM29706	lung	Johns Hopkins	430 2.0
GSM29707	lung	Johns Hopkins	430 2.0
GSM29708	lung	Johns Hopkins	430 2.0
GSM21417	lung	CNMC	430B
GSM21419	lung	CNMC	430B
GSM21421	lung	CNMC	430B
GSM21423	lung	CNMC	430B
GSM21425	lung	CNMC	430B
GSM21427	lung	CNMC	430B
GSM21404	lung	CNMC	430B
GSM21406	lung	CNMC	430B
GSM21408	lung	CNMC	430B
GSM21410	lung	CNMC	430B
GSM21412	lung	CNMC	430B
GSM21414	lung	CNMC	430B
GSM31065	muscle	Kaminski	430 2.0
GSM31114	muscle	Kaminski	430 2.0
GSM31115	muscle	Kaminski	430 2.0
GSM31116	muscle	Kaminski	430 2.0
GSM31118	muscle	Kaminski	430 2.0
GSM31121	muscle	Kaminski	430 2.0
GSM31125	muscle	Kaminski	430 2.0
GSM31128	muscle	Kaminski	430 2.0
GSM31129	muscle	Kaminski	430 2.0
GSM31130	muscle	Kaminski	430 2.0
GSM31131	muscle	Kaminski	430 2.0
GSM31132	muscle	Kaminski	430 2.0
GSM31133	muscle	Kaminski	430 2.0

GSM31134	muscle	Kaminski	430 2.0
GSM31135	muscle	Kaminski	430 2.0
GSM31136	muscle	Kaminski	430 2.0
GSM31137	muscle	Kaminski	430 2.0
GSM31138	muscle	Kaminski	430 2.0
GSM53236	muscle	Burden	430 2.0
GSM53237	muscle	Burden	430 2.0
GSM53238	muscle	Burden	430 2.0
GSM53239	muscle	Burden	430 2.0
GSM16840	muscle	Kaminski	430A
GSM16841	muscle	Kaminski	430A
GSM16842	muscle	Kaminski	430A
GSM16843	muscle	Kaminski	430A
GSM16844	muscle	Kaminski	430A
GSM16845	muscle	Kaminski	430A
GSM16846	muscle	Kaminski	430A
GSM16847	muscle	Kaminski	430A
GSM16848	muscle	Kaminski	430A
GSM16849	muscle	Kaminski	430A
GSM16850	muscle	Kaminski	430A
GSM16851	muscle	Kaminski	430A
GSM16852	muscle	Kaminski	430A
GSM16853	muscle	Kaminski	430A
GSM16854	muscle	Kaminski	430A
GSM16963	muscle	Kaminski	430A
GSM16964	muscle	Kaminski	430A
GSM16965	muscle	Kaminski	430A
GSM16855	muscle	Kaminski	430A
GSM16856	muscle	Kaminski	430A
GSM16857	muscle	Kaminski	430A
GSM16858	muscle	Kaminski	430A
GSM16859	muscle	Kaminski	430A
GSM16860	muscle	Kaminski	430A
GSM16861	muscle	Kaminski	430A
GSM16862	muscle	Kaminski	430A
GSM16863	muscle	Kaminski	430A
GSM16957	muscle	Kaminski	430A
GSM16958	muscle	Kaminski	430A
GSM16959	muscle	Kaminski	430A
GSM16960	muscle	Kaminski	430A
GSM16961	muscle	Kaminski	430A
GSM16962	muscle	Kaminski	430A
GSM16995	muscle	Kaminski	430A
GSM17004	muscle	Kaminski	430A
GSM17005	muscle	Kaminski	430A
GSM86663	neurospheres	Slack	430A
GSM86665	neurospheres	Slack	430A
GSM86667	neurospheres	Slack	430A
GSM86669	neurospheres	Slack	430A
GSM86671	neurospheres	Slack	430A
GSM86673	neurospheres	Slack	430A
GSM86675	neurospheres	Slack	430A

GSM86664	neurospheres	Slack	430B
GSM86666	neurospheres	Slack	430B
GSM86668	neurospheres	Slack	430B
GSM86670	neurospheres	Slack	430B
GSM86672	neurospheres	Slack	430B
GSM86674	neurospheres	Slack	430B
GSM86676	neurospheres	Slack	430B
GSM44309	neurospheres	Rudnicki	430A
GSM44311	neurospheres	Rudnicki	430A
GSM44313	neurospheres	Rudnicki	430A
GSM44310	neurospheres	Rudnicki	430B
GSM44312	neurospheres	Rudnicki	430B
GSM44314	neurospheres	Rudnicki	430B
GSM44979	T cells	Rudensky	430 2.0
GSM44982	T cells	Rudensky	430 2.0
GSM44980	T cells	Rudensky	430 2.0
GSM44981	T cells	Rudensky	430 2.0
GSM95590	T cells	Rudensky	430 2.0
GSM95591	T cells	Rudensky	430 2.0
GSM92588	T cells	Davis	430 2.0
GSM92589	T cells	Davis	430 2.0
GSM26964	T cells	Sloan Kettering	430 2.0
GSM26965	T cells	Sloan Kettering	430 2.0
GSM26966	T cells	Sloan Kettering	430 2.0
GSM26967	T cells	Sloan Kettering	430 2.0
GSM26968	T cells	Sloan Kettering	430 2.0
GSM26969	T cells	Sloan Kettering	430 2.0
